# Phenothiazine: A Promising Core for Perovskite and Dye-Sensitized Solar Cells

**DOI:** 10.3390/molecules31091431

**Published:** 2026-04-26

**Authors:** Luis Alberto Illicachi, David Oliveros Garavito, Viviana Cuartas, Alberto Insuasty

**Affiliations:** 1 Research Group of Chemical and Biotechnology, Faculty of Basic Sciences, Universidad Santiago de Cali, Cali 760035, Colombia; david.oliveros01@usc.edu.co; 2 Faculty of Engineering and Administration, Universidad Nacional de Colombia, Palmira 763533, Colombia; vcuartasg@unal.edu.co; 3 Heterocyclic Compounds Research Group, Department of Chemistry, Universidad del Valle, Cali 760032, Colombia; alberto.insuasty@correounivalle.edu.co

**Keywords:** phenothiazine, perovskite solar cells, dye-sensitized solar cells, organic materials, electroactive units

## Abstract

Photovoltaic technologies represent an increasingly relevant alternative for developing renewable energy sources, particularly those based on light-harvesting materials such as perovskite solar cells (PSCs) and dye-sensitized solar cells (DSSCs), which have achieved efficiencies of 27.3% and 13.0%, respectively. In this context, phenothiazine (PTZ) has attracted considerable interest as a structural block due to its outstanding structural and photophysical properties, which also represent low production costs and reduced environmental impact. This review presents recent advances in the design and development of phenothiazine-based organic materials for photovoltaic applications, analyzing the main synthetic routes for obtaining this nucleus, as well as the fundamental aspects related to the operation of solar cells, including relevant device parameters. Furthermore, several studies focused on the synthesis, characterization, and performance of new phenothiazine-derived molecules used in photovoltaic devices are also examined. Finally, the most relevant conclusions are discussed, and future perspectives for the use of these materials in solar technologies are proposed.

## 1. Introduction

In recent decades, the increase in fossil fuel consumption has caused irreversible damage to biodiversity, the environment, and the sustainability of our planet’s natural resources. Therefore, to address these challenges, the development of alternative renewable energy sources to replace fossil fuels is of great interest. Among these alternative energy sources, solar energy is particularly attractive due to its abundance, ease of access, and clean nature. In this regard, converting sunlight into electrical energy is essential for economic growth without negative environmental impacts [1,2,3,4,5]. In this context, two technologies that stand out for harnessing sunlight as an energy source are dye-sensitized solar cells (DSSCs) and perovskite solar cells (PSCs). The former relies on dyes that capture sunlight by mimicking photosynthesis, then inject electrons into a semiconductor to generate a steady flow of electrons, thereby producing electrical energy [6,7]. In PSCs, perovskite serves as the photosensitizer, but it requires molecules capable of extracting and transporting the charge carriers generated upon irradiation; therefore, the use of organic hole transporters is necessary to improve device efficiency [8,9,10]. In this regard, the search for new compounds that can act as dyes for DSSCs or as charge-carriers for PSCs is of great importance for the development of solar photovoltaic technology.

**Figure 1:**
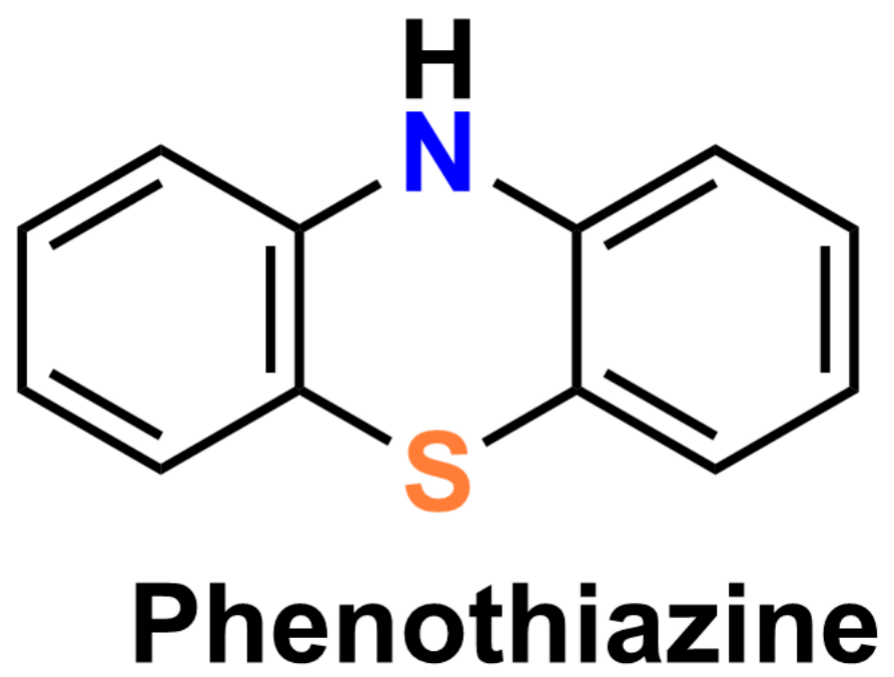
Molecular structure of phenothiazine.

Phenothiazine is a hetero-tricyclic compound that exhibits outstanding electron-donating character due to its electron-rich nitrogen (N) and sulfur (S) atoms (Figure 1) [11,12]. Phenothiazines possess a distinctive non-planar butterfly-shaped structure that prevents π–π stacking, thereby improving performance in solar cell devices by reducing undesired excimer formation [13,14]. This heterocyclic compound also exhibits a low oxidation potential and favorable photophysical properties that can be tuned by structural functionalization. Phenothiazines usually undergo electrophilic aromatic substitution reactions at positions 3 and 7, nucleophilic substitutions on the nitrogen atom, and oxidations on the sulfur atom [15,16,17].

Due to their remarkable electronic properties, phenothiazine has been used as a scaffold for the development of new organic or organometallic materials employed in perovskite solar cells (PSCs) and dye-sensitized solar cells (DSSCs), achieving record conversion efficiencies of 25.8% for PSCs [18] and 12.8% for DSSCs [19]. Herein, this review focuses on the main synthetic approaches for obtaining phenothiazine derivatives, and their use in perovskite solar cells and dye-sensitized solar cells, along with some perspectives of phenothiazine as a promising unit to develop new efficient materials.

## 2. Synthesis of Phenothiazine-Based Derivatives

**Scheme 1:**
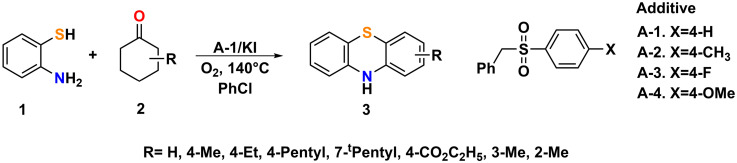
Synthesis of phenothiazine-based compounds from cyclohexanones.

Phenothiazine was synthesized for the first time by Heinrich August Bernthsen after mixing diphenylamine with sulfur at 250 °C (in a moderate 40% yield). Over the years, various synthetic routes have been developed; for instance, Liao and co-workers reported the synthesis of phenothiazine **3** from substituted 2-aminobenzenethiols and cyclohexanones via a metal-free catalytic approach (Scheme 1). They studied the effect of various organic additives, such as benzyl phenyl sulfone (**A-1**), benzyl *p*-tolyl sulfone (**A-2**), benzyl 4-fluorophenyl sulfone (**A-3**), and benzyl 4-methoxyphenyl sulfone (**A-4**), and observed higher yields with the organic additive **A-1**. Furthermore, when studying the effect of inorganic additives containing halides such as potassium bromide (KBr), potassium iodide (KI), or molecular iodine (I_2_), the yield increased up to 87% with KI. These reactions were carried out in the presence of molecular oxygen as a proton acceptor and chlorobenzene as a solvent (Scheme 1) [20].

**Scheme 2:**
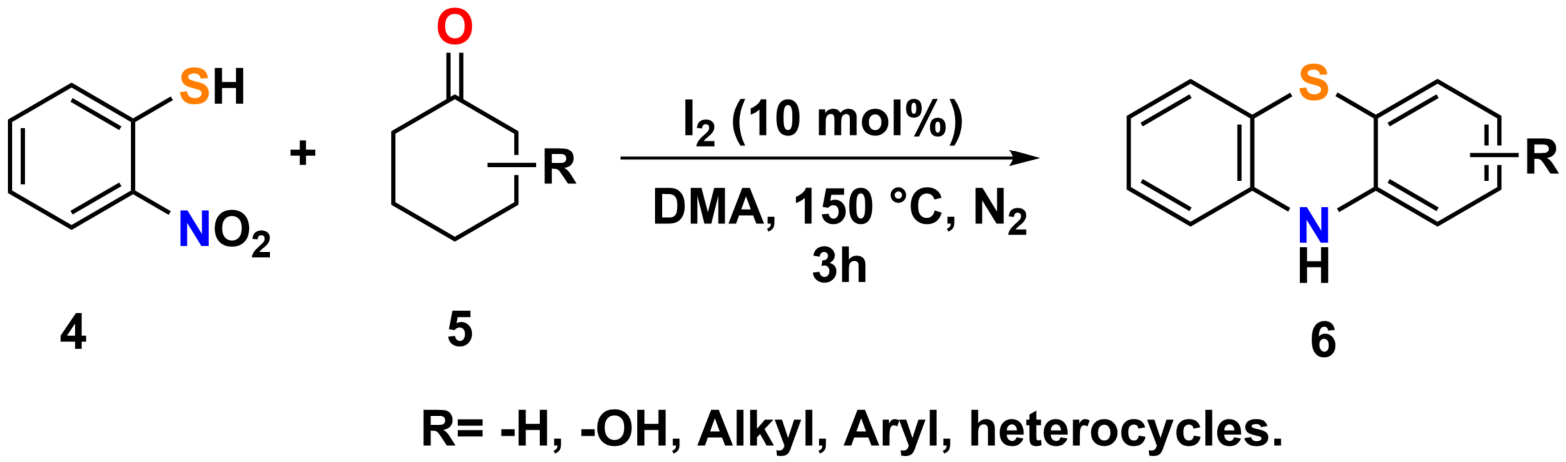
Synthesis of phenothiazine-based compounds from o-nitrothiophenols.

Zhao et al. studied the *o*-nitrothiophenols as starting materials in the synthesis of phenothiazines **6** using different catalysts such as Pd/C, Pd/Al_2_O_3_, Pd(OAc)_2_/Xantphos, Pd(TFA)_2_/(*N*,*N*-dimethylpyridin-2-amine), Pd(TFA)_2_dpephos, and I_2_, exploring the direct cyclization using molecular iodine instead of HI for the reduction in *o*-nitrothiophenols, achieving yields of 80%. Subsequently, they optimized the reaction by using acidic or basic additives, finding that the basic additives completely prevented the transformation, whereas additives such as TsOH or HOAc had little effect. Finally, they explored the use of a series of *ortho*-, *meta*-, and *para*-substituted cyclohexanones with electron-donating or electron-withdrawing groups such as alkyl, aryl, cyano, ester carbonyl, ketone carbonyl, and imide, which allowed them to obtain a wide range of substituted phenothiazines with yields between 35 and 95% (Scheme 2) [21].

**Scheme 3:**
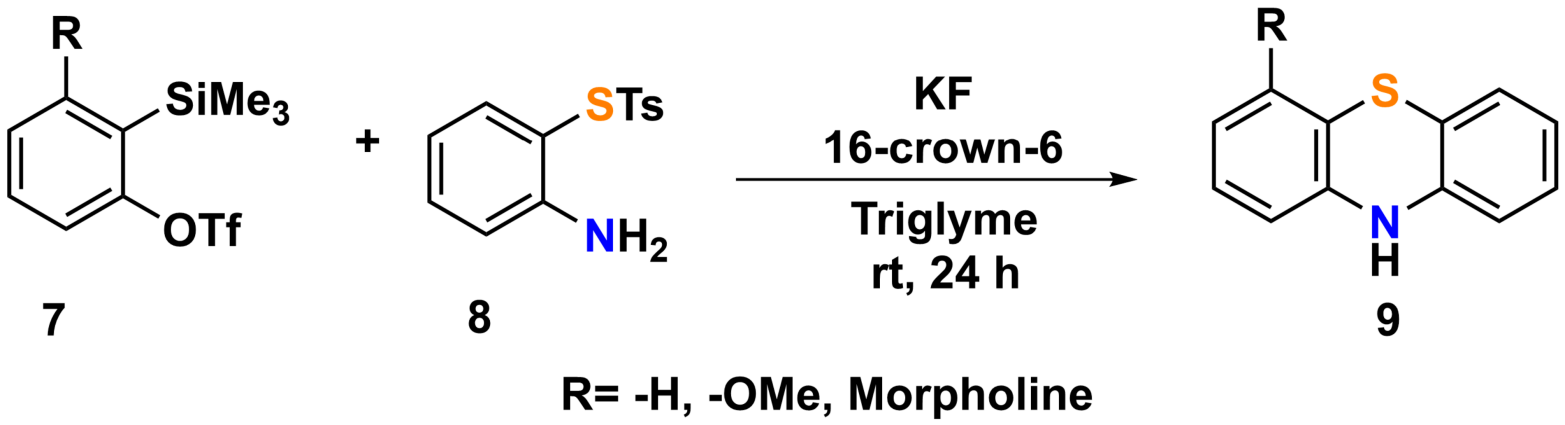
Synthesis of phenothiazine-based compounds by aryne reaction.

The use of arynes for the synthesis of heterocycles has been studied to obtain phenothiazine and phenoxazine derivatives **9**. Kanemoto’s group employed *o*-silylaryl triflates with amino/hydroxy substituted sufonates, analyzing activators like cesium fluoride (CsF), cesium carbonate (Cs_2_CO_3_), and potassium fluoride (KF) in the presence of 18-crown-6 and tetra-n-butylammonium fluoride trihydrate (Bu_4_NF·3H_2_O), finding that the optimal activator was KF, affording a phenothiazine derivative in a 60% yield. On the other hand, the effects of the solvents were also evaluated using KF, and it was found that the 1,2-bis(2-methoxyethoxy)ethane (triglyme) increased the yield up to 75%. These conditions were also extrapolated to afford yields between 64 and 84% (Scheme 3) [22].

**Scheme 4:**
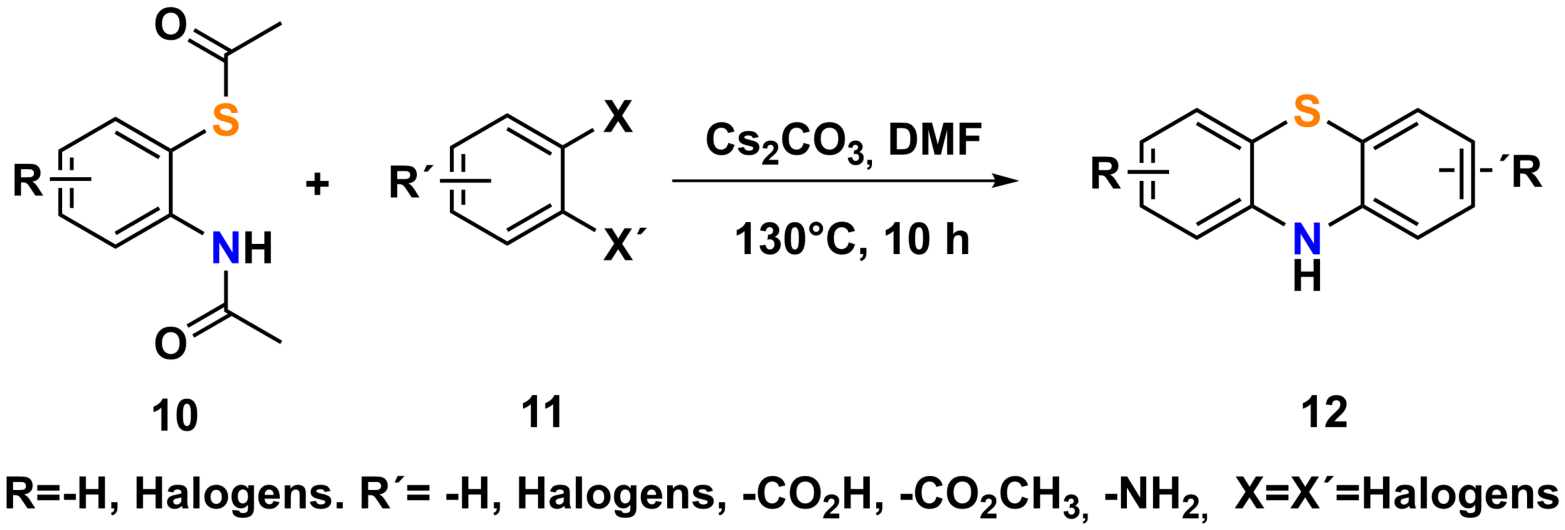
Synthesis of phenothiazines from *S*-2-acetamidophenyl ethanethioates.

Another interesting synthetic approach was performed by Zhou’s group, who carried out the transition-metal-free synthesis of phenothiazines **12** from *S*-2-acetamidophenyl ethanethioates and *ortho*-dihaloarenes (Scheme 4). Initially, they tested the *S*-2-acetamidophenyl ethanethioate and *o*-bromoiodobenzene in DMF with CsCO_3_ as a base, yielding 81%. Examining the reaction conditions, they found that the cesium carbonate significantly improved the yield compared to potassium carbonate (72% yield), and that strong nucleophilic bases such as KOH gave lower yields than non-nucleophilic bases such as potassium *tert*-butoxide, because KOH could cause saponification of reagent **10**. On the other hand, the use of aprotic polar solvents yielded worse results compared to DMF. Finally, they explored the use of different substituents in both reagents, obtaining yields of 42–91%. This presents a simple, easy-to-operate, ligand-free, and transition metal-free alternative for the synthesis of phenothiazines [23].

**Scheme 5:**
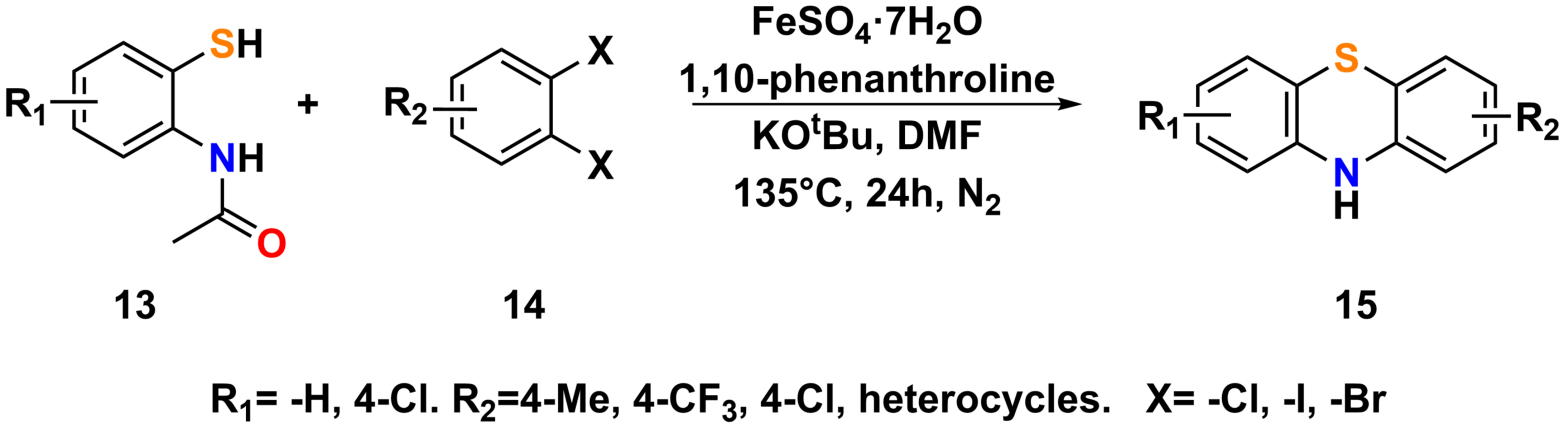
Synthesis of phenothiazine derivatives via a domino iron-catalyzed C–S/C–N cross-coupling reaction.

Moreover, transition metal catalysis has been widely studied, employing iron, palladium, nickel, platinum, rhodium, and copper to present innovative alternatives with high performance and low environmental impact. In this regard, Hu and Zhang reported the synthesis of phenothiazines **15** via cross-coupling reactions employing different iron salts as catalysts, such as iron sulfate heptahydrate (FeSO_4_·7H_2_O), iron nitrate (Fe(NO_2_)_3_·9H_2_O), and iron (III) chloride (FeCl_3_) [24]. The reactions were performed using 1,10-phenanthroline, potassium *tert*-butoxide (KO*t*Bu), and DMF as the solvent, affording the desired compounds with yields around 70%. Also, when studying the use of different 1,2-dihalobenzenes and quinoxaline dihalides, it was found that yields improved significantly with 1-chloro-2-iodobenzene and 1-bromo-2-chlorobenzene, compared with 1-bromo-2-iodobenzene or 1,2-dibromobenzene, which reported yields higher than 90% (Scheme 5).

**Scheme 6:**
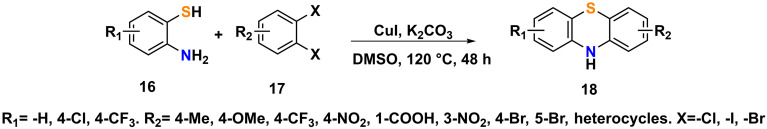
Synthesis of phenothiazine-based compounds via ligand-free CuI-catalyzed catalysis.

A copper-catalyzed approach has also been explored for the synthesis of phenothiazines (**18**), in which various copper salts have been evaluated, including copper iodide (CuI), copper chloride (CuCl), and copper bromide (CuBr), finding that the CuI combined with potassium carbonate (K_2_CO_3_) resulted in yields higher than 70% when starting from substituted *o*-dihalobenzenes and 2-aminobenzenethiols. Moreover, it was observed that the use of 1-bromo-2-chlorobenzene or 1-bromo-2-iodobenzene improved the yields to 86% and 91%, respectively (Scheme 6), making this alternative ligand-free [25]. Copper salts are not the only method for this type of synthesis; also, the copper oxide supported on poly-ionic resins (CuO@ARF) was reported, observing that the catalyst could be reused up to five times without significantly decreasing yield under moderate conditions, unlike palladium or platinum catalysts, which require an inert atmosphere or anhydrous solvents [26].

**Scheme 7:**
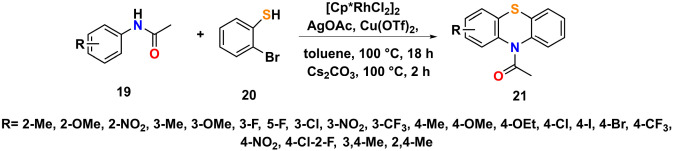
Synthesis of phenothiazine-based compounds via Rhodium-catalyzed catalysis.

Rui et al. [27] investigated the C-H thiolation using rhodium, ruthenium, palladium, and copper catalysts, reporting the synthesis of phenothiazine and derivatives **21** through C-S bond formation. They evaluated different catalysts such as copper acetate (Cu(OAc)_2_), palladium acetate (Pd(OAc)_2_), pentamethylcyclopentadienylrhodium(III) chloride dimer ([Cp*RhCl_2_]_2_) and dichloro(*p*-cymene)ruthenium(II) dimer (RuCl_2_(*p*-cymene)), finding that the optimum catalyst was ([Cp*RhCl_2_]_2_) using AgOAc as oxidant and copper(II) trifluoromethanesulfonate (Cu(OTf)_2_) as additives (Scheme 7), resulting in yields around 92%.

## 3. Perovskite Solar Cells (PSCs)

**Figure 2:**
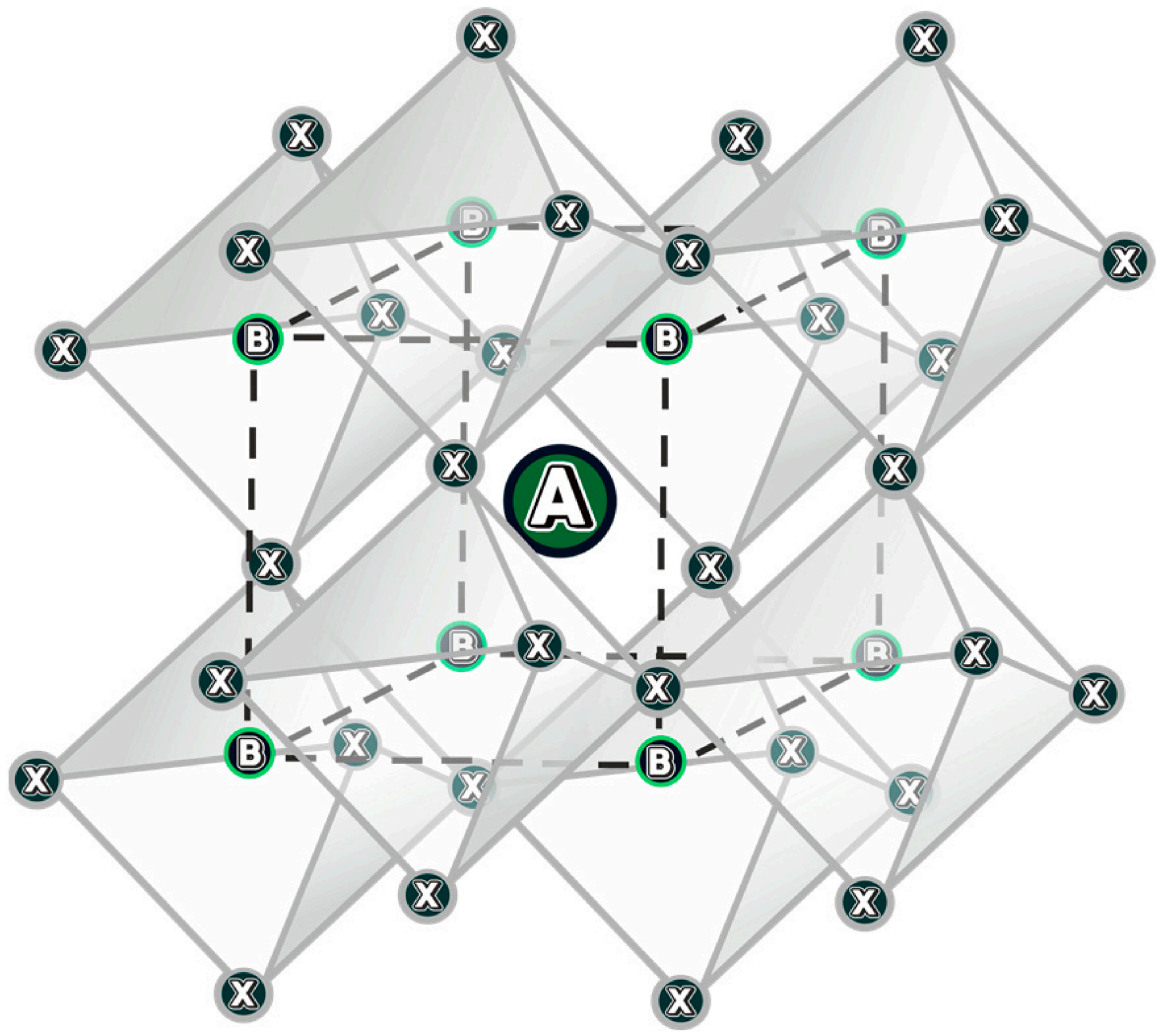
Crystal structure of perovskite materials.

Perovskite-based solar cells (PSCs) have been presented as an attractive alternative to crystalline silicon cells. PSCs are part of the third generation of photovoltaic (PV) technologies, with a power conversion efficiency (PCE) of 27.3% for single junction solar cells [28], exhibiting excellent photoelectronic properties such as absorption coefficient, charge carrier mobility, dielectric constant, and bipolar characteristics, comparable to first-generation cells such as crystalline silicon cells [29]. Perovskite, named after the Russian mineralogist Lev Perovski, is a family of compounds with a specific crystal structure with the formula ABX_3_ [30]. The crystal structure is shown in Figure 2, where A (A = MA^+^(CH_3_NH_3_^+^), FA^+^(CH=NH_2_^+^), Cs^+^) corresponds to a cation with a cube-octahedral occupation, while cation B (B = Pb^2+^, Sn^2+^, among other transition metals and metalloids) is the center of an octahedron surrounded by six anions X^−^; these anions are mainly halogens (Cl^−^, Br^−^, I^−^) [31].

**Figure 3:**
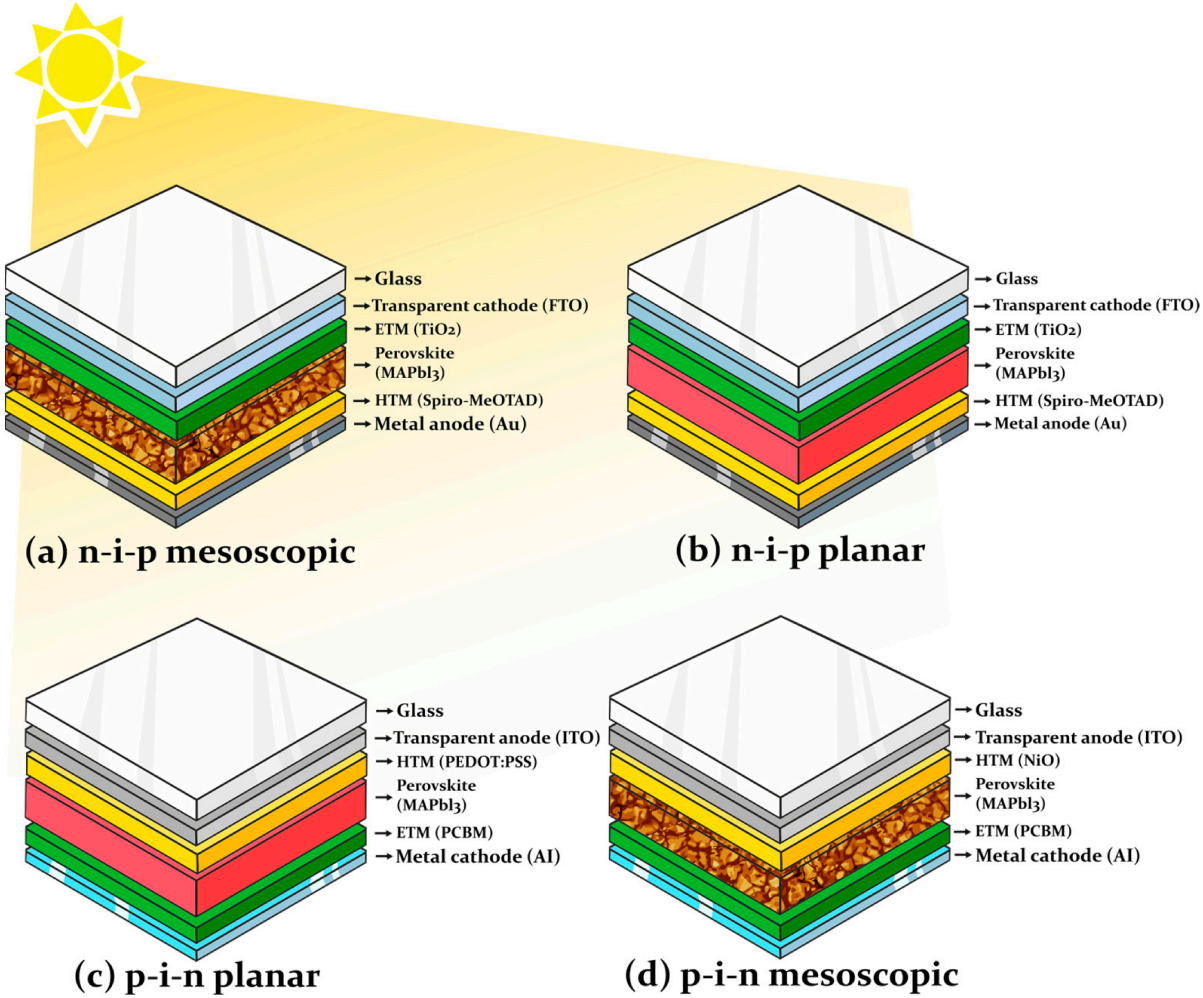
Types of perovskite solar cells and their classification according to the deposition of the layers.

PCSs have two main structures classified according to the deposition of the layers: conventional (n-i-p) (Figure 3a,b) and inverted (p-i-n) (Figure 3c,d), which are in turn divided into two categories: mesoscopic and flat. Mesoscopic cells incorporate a mesoporous layer, while flat cells have fewer surface imperfections [32]. Conventional PCS (n-i-p) have an architecture consisting of a transparent glass cathode coated with a transparent conductive oxide (TCO) followed by electron transport material (ETM), then perovskite, followed by a hole transport material (HTM), and finally, the metal anode, while inverted cells, as the name suggests, completely invert the layers of the conventional cell. Mesoscopic cells use metal oxides (TiO_2_ or ZrO_2_) (Figure 3a,d) as an electron transport layer (ETL), especially in dye-sensitized solar cells, which are the origin of solution-processable PSCs. They also offer advantages such as high PCE, low-cost production materials, and simple manufacturing processes, although the morphology of the mesoporous layer is crucial for modifying device performance [33,34,35,36]. On the other hand, flat heterojunction PSCs are characterized by the absorption of the perovskite film sandwiched between a layer of HTM and ETM, and the respective electrodes (Figure 3b,c) [37].

**Figure 4:**
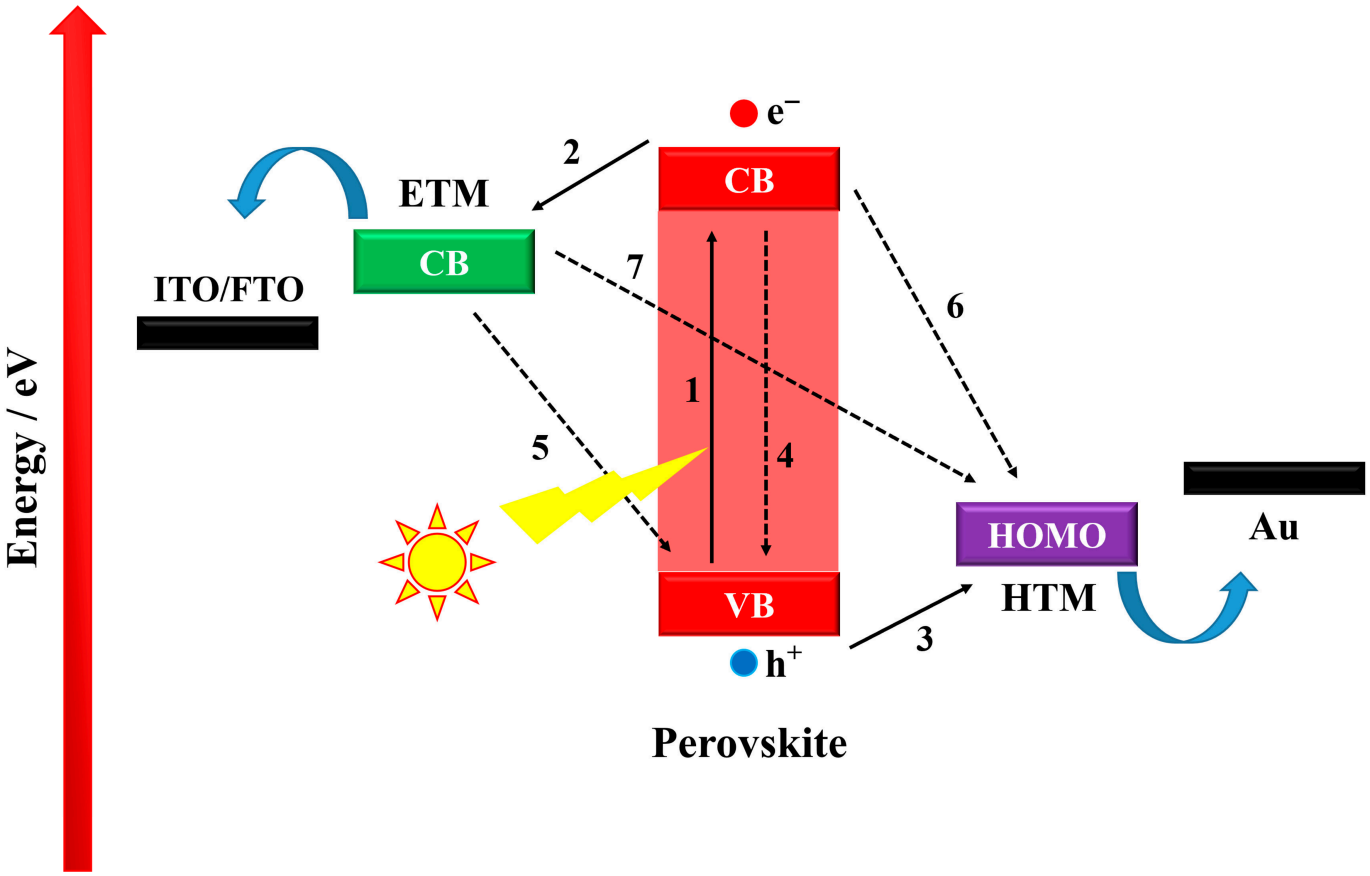
Working principles of PSCs.

Regarding the operation of PSC devices (Figure 4), it has been determined that, upon illumination, the perovskite layer generates an electron–hole pair (exciton) (1). This pair is weakly bound due to the exciton’s low binding energy (2 to 55 meV). The electrons and holes produced are extracted by the n-type electron carrier (2) and the p-type hole carrier (3), respectively [38,39]. Notably, the perovskite layer is always located between the HTM and the ETM, regardless of device architecture. Both the HTM and the ETM serve as transport layers and as protective layers against oxygen and moisture. As a result, ETMs and HTMs play a crucial role in determining the performance and stability of the fabricated device. On the other hand, undesirable processes in cell operation include the recombination of photogenerated charge carriers (4), reverse electron transfer at the TiO_2_–perovskite interface (5), reverse hole transfer at the HTM–perovskite interface (6), and charge recombination at the TiO_2_–HTM interface if the perovskite layer coverage is incomplete. To achieve high efficiencies, processes 4–7 must occur on timescales longer than those of the generation, separation, and extraction processes 1–3 [40,41].

The efficiency of a solar cell depends on the appropriate selection of all components that optimize both kinetics and energy efficiency. The PCE hangs on several parameters such as (1) the short-circuit current density (*J_sc_*), which is the maximum current density in the cell when the applied voltage is zero; (2) the open-circuit potential (*V_oc_*), which is the maximum voltage available from the cell when there is no current; and (3) the fill factor (*FF*), which is the ratio between the maximum power of the cell and the product of *V_oc_* and *J_sc_*, with values between zero and one reflecting electrical and electrochemical losses during operation of the cell. To achieve high energy conversion efficiency, the highest possible values of *V_oc_*, *J_sc_*, and *FF* are required [42].

### Phenothiazine in PSCs

**Scheme 8:**
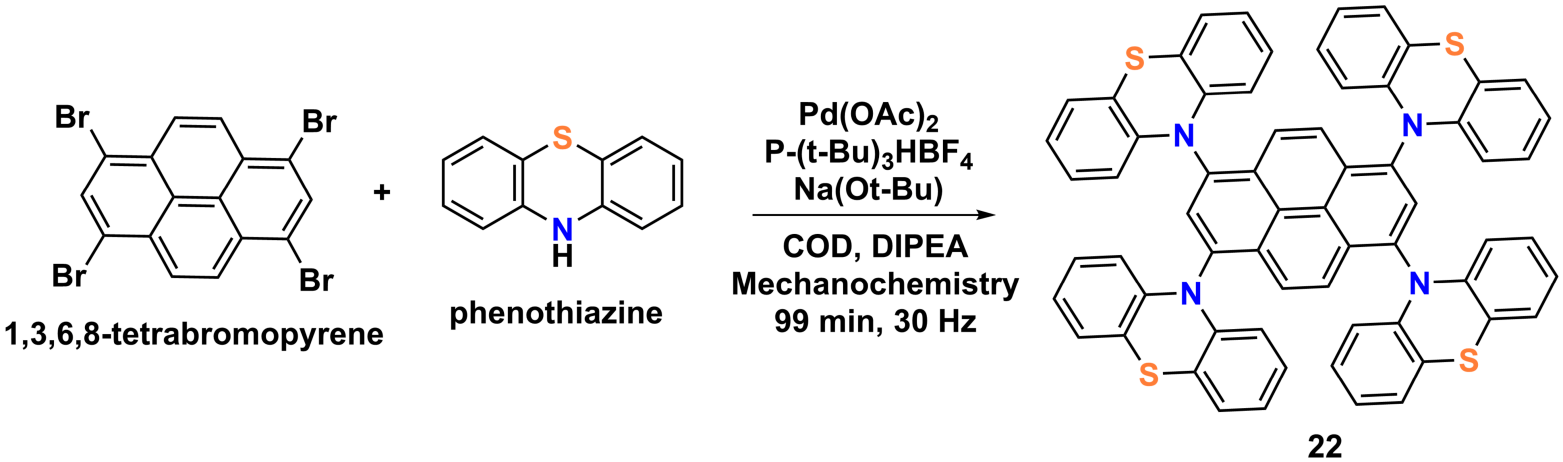
Preparation of pyrene–phenothiazine hybrids by mechanochemistry.

Owing to its remarkable photophysical, structural, and electronic properties, phenothiazine is a very attractive scaffold for the development of new hole-transport materials (HTMs). In this section, we present the most recent studies conducted in PSCs in which phenothiazine plays a key role in the design of electroactive materials. Dhokale and collaborators [43] afforded a four-fold C-N bond formation between 1,3,6,8-tetrabromopyrene and phenothiazine (Scheme 8), which was performed by a mechanochemical approach under ambient conditions and air-stable reagents without the need for a Schlenk line or glovebox, affording the desired phenothiazine-based pyrene **22** in good yield, reporting an alternative and efficient technique for the synthesis of HTMs. The obtained material presented HOMO/LUMO values of −5.01 and −2.31 eV, respectively, as well as an absorption range between 300 and 380 nm, with a wavelength of maximum absorption of 359 nm in solution with toluene, while the emission spectrum presents the main band around 600 nm in toluene, 622 nm in chloroform, and 658 nm in dichloromethane. The bathochromic effect of emission maxima with solvent polarity is due to charge transfer character from phenothiazine to pyrene. The performance of n-i-p PSCs architecture with the configuration indium tin oxide (ITO)/ETL/absorbing layer (perovskite)/HTL(22)/Au was evaluated for both unencapsulated and encapsulated devices. Unencapsulated devices (i) initially showed a *J*_sc_ of 14.28 mA·cm^−2^, *V*_oc_ of 0.95 V, and *FF* of 71.63 ± 1.69%, resulting in a PCE of 10.61 ± 1.24%, which, after four weeks, decreased to *J*_sc_ of 11.56 mA·cm^−2^, *V*_oc_ of 0.70 V, and *FF* of 46.33 ± 1.84% (PCE of 4.09 ± 1.37%). In contrast, the encapsulated PSCs (ii) initially yielded *J*_sc_ of 14.32 mA·cm^−2^, *V*_oc_ of 0.95 V, and *FF* of 72.86 ± 1.36%, corresponding to a PCE of 10.83 ± 1.62% at the beginning. Then, it decreased to a *J*_sc_ of 12.26 mA·cm^−2^, *V*_oc_ of 0.94 V, and *FF* of 70.11 ± 1.63%, corresponding to a PCE of 8.83 ± 1.73%. This drastic decrease is attributed to the degradation of the perovskite layer beneath the HTL due to exposure to the open atmosphere.

**Figure 5:**
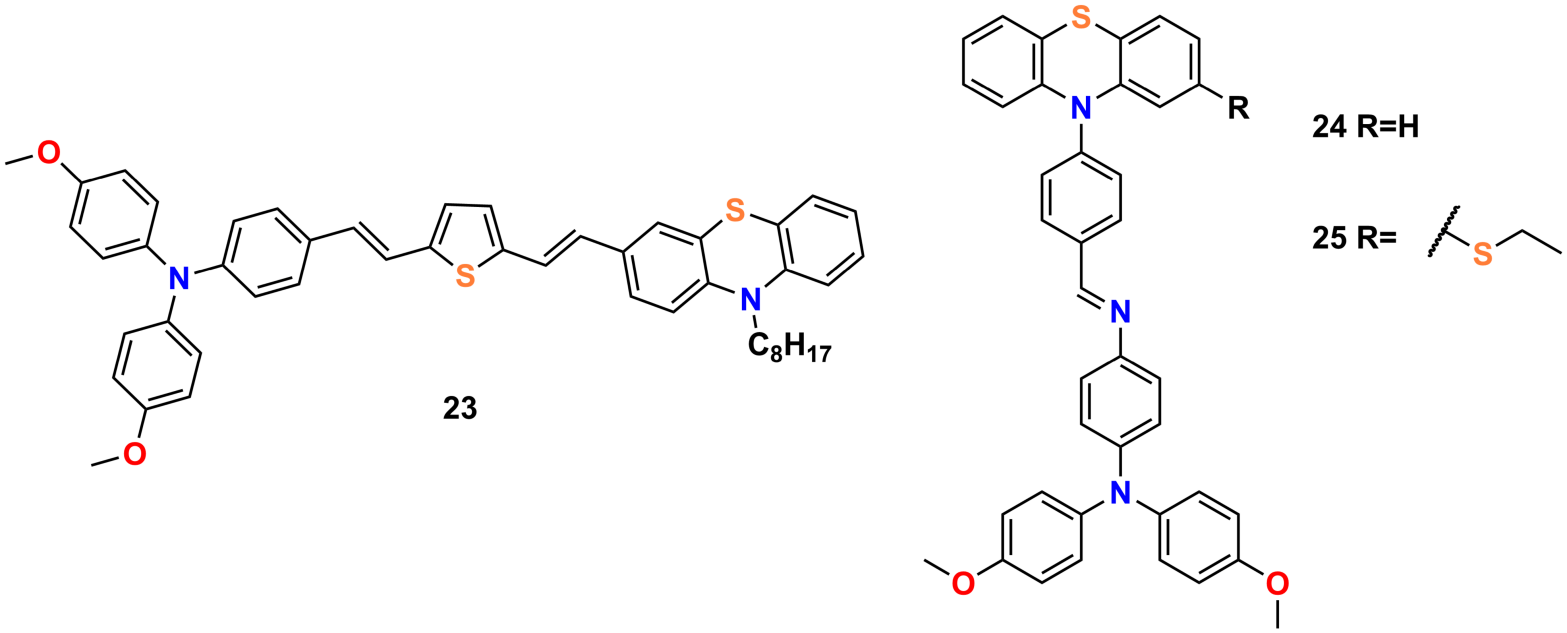
Chemical structure of triphenylamine–phenothiazine derivatives used as HTMs for PSCs.

Ganesan et al. synthesized a new vinylene–thiophene–vinylene moiety linked to triphenylamine–phenothiazine **23** as an unsymmetrical D-π-D small molecule (Figure 5). The new material exhibited a wavelength of maximum absorption at 447 nm and emission at 544 nm, being associated with the π-π* transitions of the molecule. The HOMO was found to be −5.2 eV lower than that of spiro-OMeTAD (−5.12 eV) but higher than that of perovskite (−5.4 eV), while the LUMO was −2.66 eV. Moreover, it was found that incorporating an interfacial layer of compound **23** significantly improved the device’s performance. Two conditions were evaluated in an n-i-p cell architecture with the configuration ITO/SnOx/FA_0_._9_Cs_0_._1_PbI_3_/**23**/spiro-OMeTAD/Ag: a control device without the modifier (0 mg/mL) and a target device with the modifier (0.1 mg/mL). The control device (i) yielded a *V_oc_* of 0.96 V, *J_sc_* of 21.52 mA·cm^−2^, *FF* of 63.40%, and a PCE of 13.09%. In contrast, the device treated with the 0.1 mg/mL concentration (ii) showed significant increases in *J_sc_* 24.57 mA·cm^−2^, *FF* 67.64%, and overall PCE (15.88%), while the *V_oc_* remained constant. The improvement in the *FF* and PCE of the device is attributed to the broad conjugation that results in the delocalization of the π electrons along the main chain of the molecule, as well as to the π-π interactions of the molecule, which decrease interfacial resistance and thus improve the filling factor. In addition, they observed that when a drop of water came into contact with the cell modified with **23** (0.1 mg/mL), an angle of 86° was formed, while in the control device, the angle was 54.6°, which can be attributed to the long alkyl chain present in the molecule, improving hydrophobicity and stability against environmental conditions, keeping the PCE stable for 280 h [44]. Salunke and co-workers synthesized two new low-cost phenothiazine-based materials from a simple and environmentally friendly procedure without the use of chlorinated solvents. This involved the condensation of an electro-donor triarylamine and phenothiazine group (**24**, Figure 5) linked by an azomethine group with the addition of a thioethyl chain at position 2 of the phenothiazine (**25**) to improve solubility and the possibility of greater HTM/perovskite interactions [45]. The introduction of a methoxy-substituted triarylamine in the system ensured a good alignment between the highest occupied molecular orbital (HOMO) levels of the HTMs and the valence band (VB) of the perovskite, with good solubility in toluene (20 mg/mL).

Compound **24** presented optical properties with an absorption maximum at 409 nm in DCM and at 412 nm in film, and emission at 565 nm in DCM and at 567 nm in film; on the other hand, compound **25** presented an absorption at 412 nm in DCM and at 417 nm in film, with an emission at 565 nm in DCM and 576 nm in film; in both cases, these bands correspond to intramolecular charge transfer from the electron-donating methoxy-substituted triarylamine to the azomethine unit. The slight shift in absorption of **25** in comparison to **24** is attributed to the presence of the electron-rich thioethyl group. Since HOMO levels correspond to the ionization potentials of these materials, both **24** and **25** exhibited similar electrochemical properties, with their HOMO levels located at −4,97 and −4,99 eV, respectively, compared to the HOMO level of Spiro-OMeTAD (−4.82 eV). The small difference in the HOMO levels of **24** (0.15 eV), **25** (0.17 eV), and Spiro-OMeTAD indicates that both HTMs are highly compatible with the triple-cation halide perovskite (Cs_0,05_MA1-yFAyPbI_3_-xClx), whose VB is −5.9 eV. Likewise, the LUMO levels of both compounds are similar, at −2.41 and −2.45 eV, respectively. On the other hand, the photovoltaic parameters obtained for HTMs showed an increase in *J*_sc_ of 19.75 and 19.83 mA·cm^−2^, *FF* of 0.49 and 0.58%, and PCE of 9.77 and 11.62% for **24** and **25**, respectively, while the obtained *V*_oc_ was 1.01 V for both HTMs. Additionally, **24** presented low hydrophobicity (water contact angle, CA = 86.6°), excellent environmental stability, and a shelf life of 6 months under environmental conditions, compared with **25**, which showed high hydrophobicity (CA = 92°).

**Figure 6:**
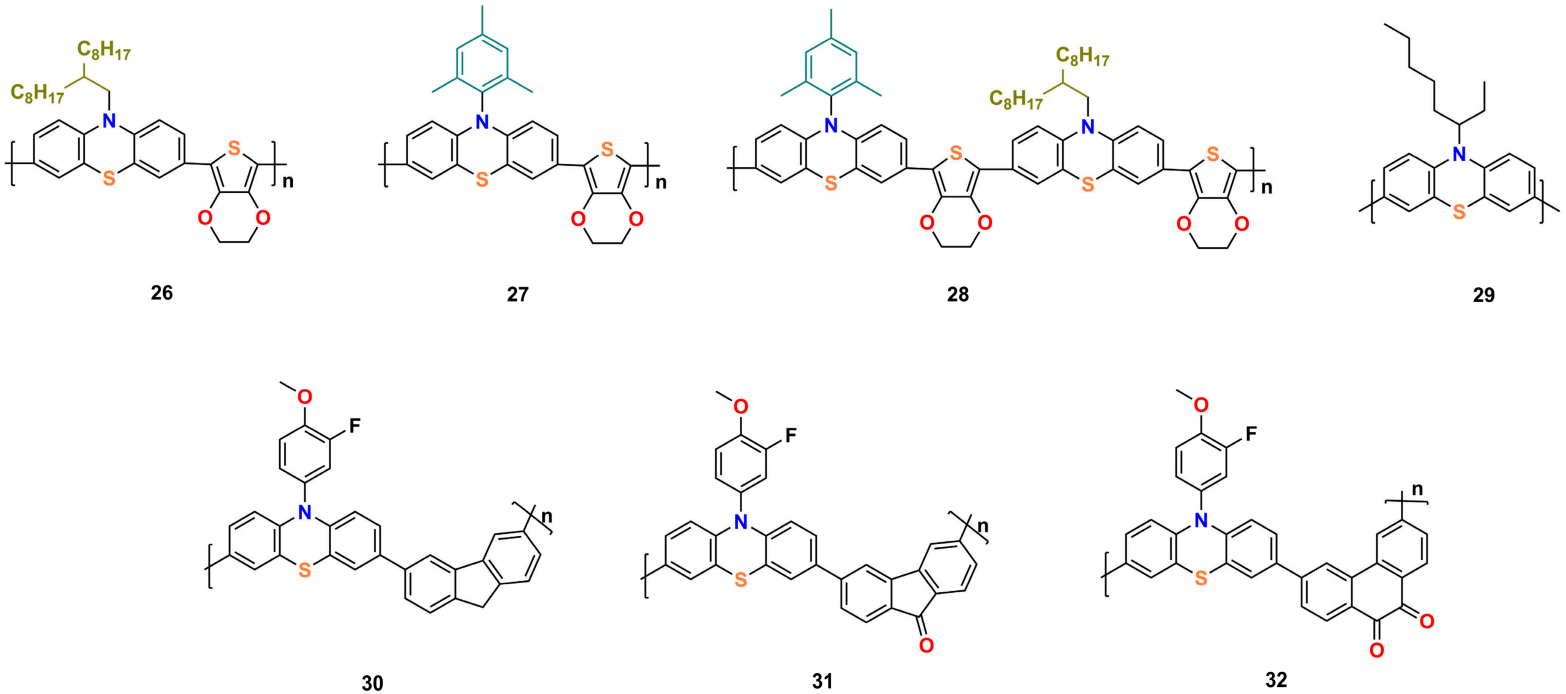
Polymers based on phenothiazine are used as HTMs in PSCs.

Zhang et al. [46] synthesized three phenothiazine–ethylenedioxythiophene polymers featuring varied substituents on phenothiazine at the *N* position: 2-octyldodecyl (**26**), trimethylphenyl (**27**), and alternating between 2-octyldodecyl and trimethylphenyl groups (**28**) (Figure 6). This substitution variation led to differences in the HOMO energy levels of the polymeric semiconductors, influencing air oxidation doping and the rate of hole extraction from photoexcited perovskite. They also found that the variation in the substituents significantly affects the PCE, varying from 19.4% with 2-octyldodecyl to 25.1% with the combination of both substituents (Table 1, entries 5–7), demonstrating an enhancement in photovoltaic properties compared with the reference material Spiro-OMeTAD. Additionally, the use of flexible and rigid substituents gives the polymer film a uniform morphological surface, improving the hole mobility in PSC with a structure of ITO/SnO_2_/FAPbI_3_/TTI-derived interlayers (triphenylmethane triisocyanate)/HTL/Au. Also, the use of alternative methodologies that do not employ noble metals to obtain polymers (**29**, Figure 6) was explored by Li et al. [47] and represents a more desirable option for the commercialization of perovskite solar cells. The use of phenothiazine in this kind of polymer serves as an energy modulator, reducing voltage loss and showing good optoelectronic performance when used in an inverted PSC (Table 1, entry 8). It exhibited high hydrophobicity and thermal stability, as well as significant efficiency retention for more than 800 h in a device of ITO/HTM/perovskite/C_60_/bathocuproine (BCP)/Ag structure, with a triple cation perovskite Cs_0,05_(FA_0.9_MA_0.1_)_0.95_Pb(I_0.9_Br_0.1_)_3_, resulting in a PCE of 21.38%, which is better than the PTAA polymer (PCE 19.8%) use as a reference. Phenothiazine-based polymers endowed with fluorene, 9-fluorenone and phenanthrenequinone as copolymer units (**30–32**, respectively, Figure 6) were reported by Tan et al., showing these structural modifications can modulate the spatial packing notably, which improves the film’s layout on the cell, reducing the structural defects and increasing the hydrophobicity with a decrease in the contact angle from 93.576° to 79.382° when the film encounters water. These modifications also increased the thermal stability of polymers with T_d_ values above 249 °C. On the other hand, a significant increase in photovoltaic parameters (Table 1, entries 9–11) may be due to the molecular flatness, with a rise from 15.3% to 17.3% in PCE, and 1.03 V to 1.10 V in *V*_oc_ [48].

**Figure 7:**
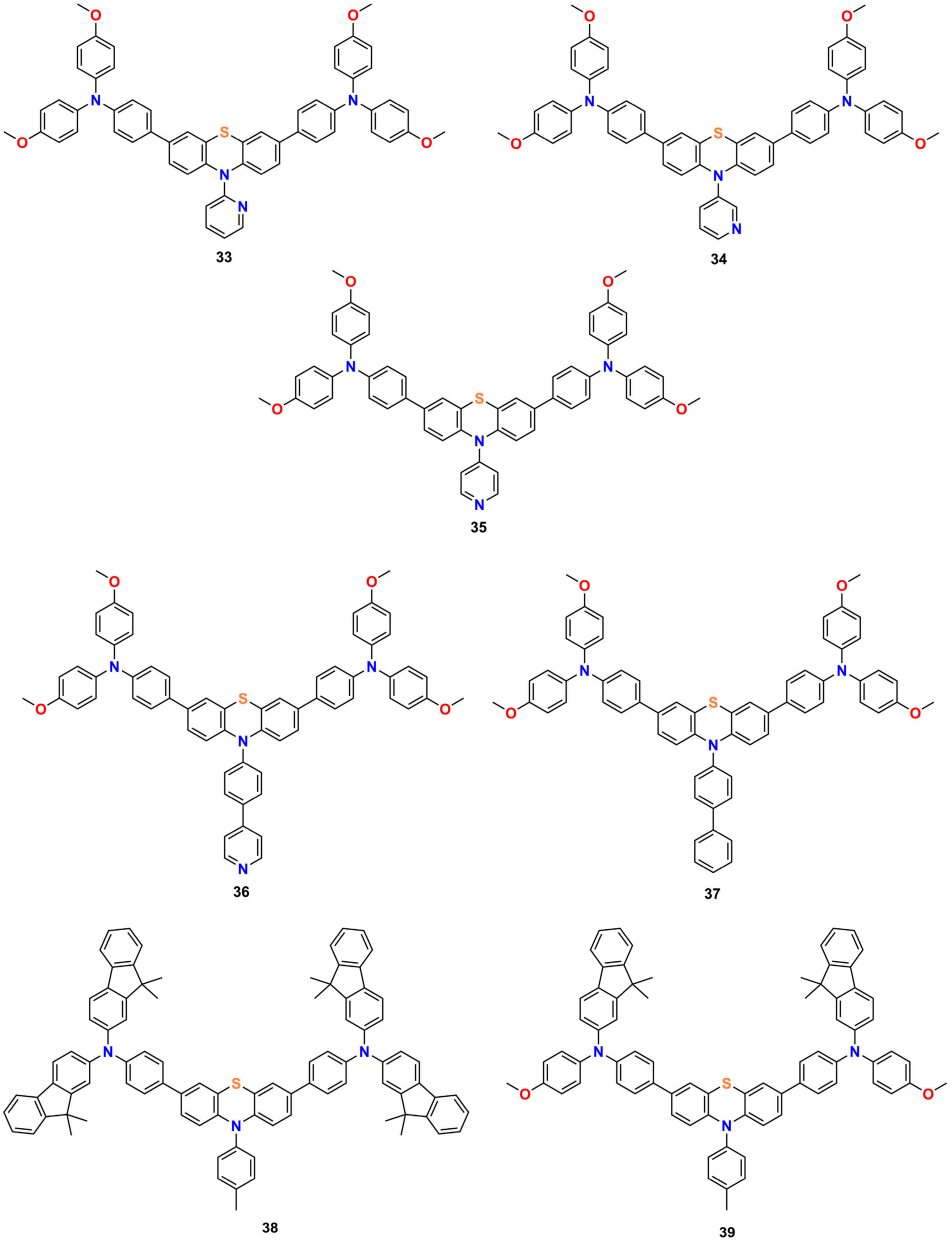
Structure of different phenothiazine derivatives used as HTMs in PSCs.

Huang and colleagues [49] studied the use of phenothiazine small molecules based on pyridine isomers (**33**–**35**) as hole-selective layers (HSL) in PSCs, which play an important role in the extraction and transport of photogenerated holes, and observed that the position of the isomer directly affects the optoelectronic properties. The absorption bands ranged from 309 to 349 nm, 309 to 334 nm, and 310 to 348 nm, for compounds **33**–**35**, respectively. Such bands are attributed to π-π* transitions and intramolecular charge transfers. In addition, the optical band gaps and the HOMO level showed gradual variations depending on the position of the isomer. They also reported high PCE values of 16.05 and 17.93% for isomers 2-(**33**) and 3-(**34**), respectively, compared to isomer 4-(**35**) with a PCE of 0.40%. On the other hand, an increase in conjugation in these systems by additional aromatic rings was studied (**36** and **37**, Figure 7), observing that the system endowed with a 4-phenylpyridine group (**36**) presented significant improvements in film formation, charge transport, and a PCE close to 20%, similar to that reported for the reference material spiro-OMeTAD (19.7%) in the backward configuration, as well as adequate energy levels with perovskite, facilitating the extraction and transport of the generated holes. Meanwhile, the addition of the diphenyl group (**37**) significantly decreased the optical and electronic properties of HTM, going from 19.9% to 13.2% PCE. The maximum absorption also shifted from 335 for **36** to 330 for **37**, as well as a decrease in conductivity from 7.19 to 7.05 × 10^−5^ S·cm^−1^, respectively. Considering the excellent hole transport properties of compound **36**, Ding and colleagues used this compound at the perovskite-Spiro-OMeTAD interface, observing that the insertion significantly improved the cell’s PCE value, increasing it from 19.7% to 22.1% [50]. Zhai’s group designed and synthesized new HTMs from *N*,*N*′-di(9,9-dimethyl-9H-fluoren)-phenylamine (**38**, Figure 7) and *N*-(4-methoxyphenyl)-*N*-(9,9-dimethyl-9H-fluoren)-phenylamine (**39**, Figure 7) to study how peripheral symmetry affects the device’s performance. The authors found that both compounds had similar optoelectronic properties, and the substitution of methoxybenzene for a fluorene unit in compound **39** showed an improvement in these properties, yielding a PCE of 20.15%, which was higher than that of the reference material, spiro-OMeTAD (18.57%). In addition, compound **39** represents a low-cost synthesis process and is a better material for fabricating a stable device than spiro-OMeTAD, maintaining 74.5% of its initial efficiency after 1000 h [51].

**Figure 8:**
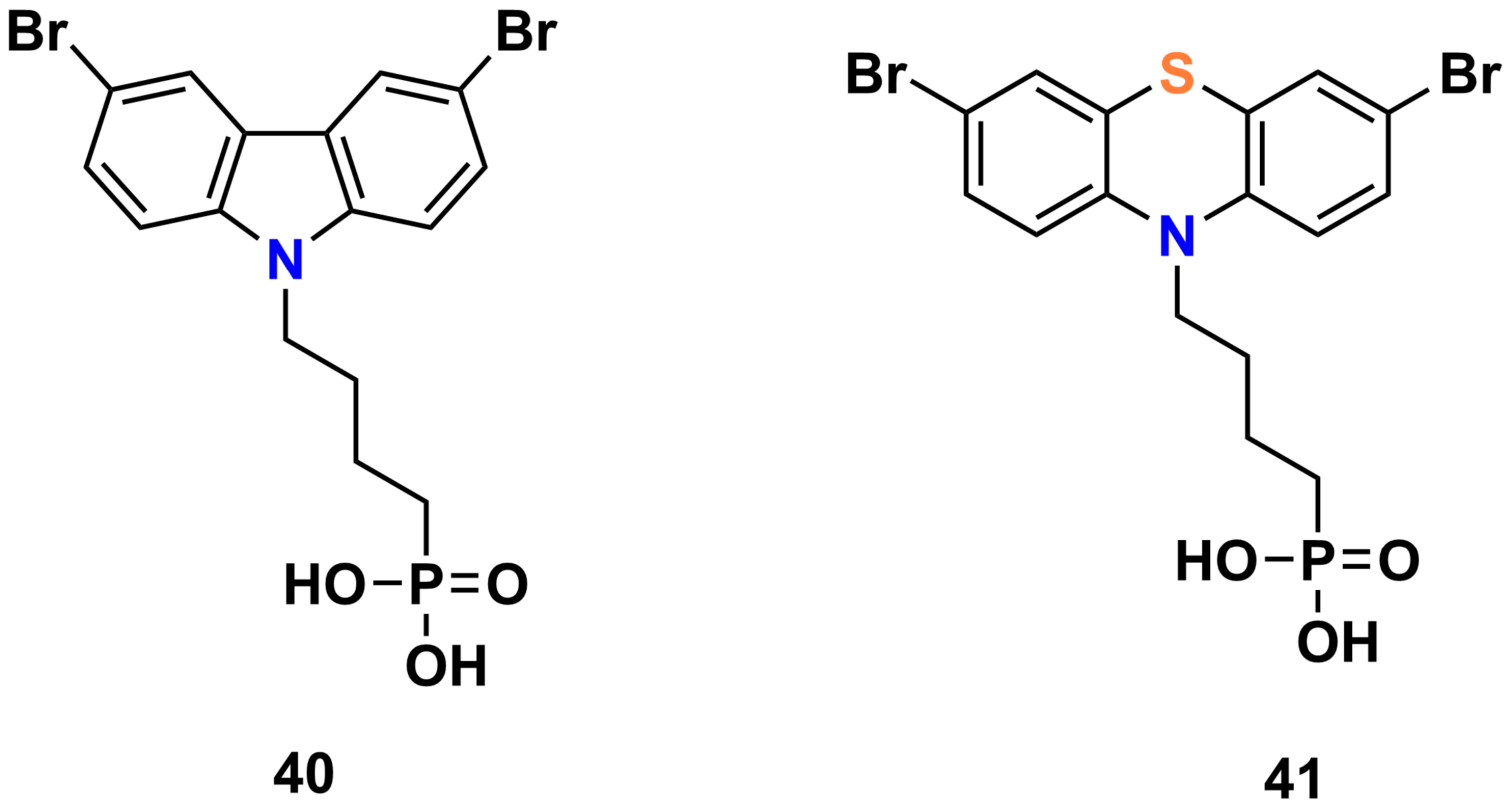
Carbazole and phenothiazine phosphoric acid-based HTMs for PSCs.

Li and colleagues [52] synthesized two dibrominated compounds, **40** and **41**, based on carbazole and phenothiazine, respectively, linked to phosphoric acid (Figure 8). Compound **40**, based on carbazole, shows a poor efficiency of 4.24% due to the unmatched interface energy level, while compound **41**, based on phenothiazine, shows a PCE of 22.06%, which is much better than the carbazole derivative. The introduction of a sulfur atom into the phenothiazine core significantly increases the electron-donating capability relative to the carbazole core; this directly affects the HOMO energy level, which is closer to the valence band of the perovskite. On the other hand, when the PCE of **41** is compared with the reference material PTAA (20.91%), it exhibited better behavior. In this case, the use of a phenothiazine core enhanced photovoltaic properties, making it a promising choice for large-scale, flexible devices in the future.

**Figure 9:**
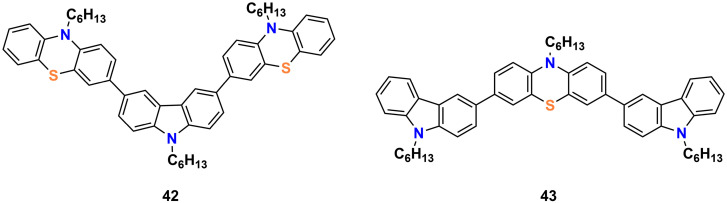
Carbazole–phenothiazine derivatives as HTMs for PSCs.

Carbazole- and phenothiazine-derived HTMs were studied by Maruzzo et al. [53], who designed and synthesized two new symmetrical molecules (**42** and **43**, Figure 9) using Suzuki coupling reactions. The compounds obtained showed maximum absorption bands in solution at 264 and 290 nm for **42** and **43**, respectively. Compound **42** had a slightly lower HOMO level (−5.33 eV) than compound **43** (−5.22 eV), as well as optoelectronic yields when using a 0.5 mg·mL^−1^ solution of a *V*_oc_ of 0.95 ± 0.04 V, *J*_sc_ 18.7 ± 0.8 mA·cm^−2^, *FF* 78.8 ± 3.2%, and PCE 14.0 ± 0.7% for compound **42**, while for compound **43**, the parameters were slightly lower at 0.83 ± 0.03 V, *J*_sc_ 15.8 + 0.5 mA·cm^−2^, *FF* 78.8 ± 3.2%, and PCE 10.4 ± 0.8%. Because of this, carbazole–phenothiazine derivatives are a promising alternative thanks to their low production costs and high optoelectronic yields in cells with the structure of ITO/HTM/Cs_0.17_FA_0.83_Pb(I_0.08_Br_0.20_)_3_/PC_61_BM/bathocuproine BCP/Cu. This work stands out because the authors demonstrate how the same starting scaffolds assembled in a different way in the final material led to distinct performances, explained by different electronic behavior. Both molecules **42** and **43** demonstrated to be suitable as promising low-cost HTMs for perovskite solar cells when implemented in inverted devices and were found to be stable under air storage after one year.

**Figure 10:**
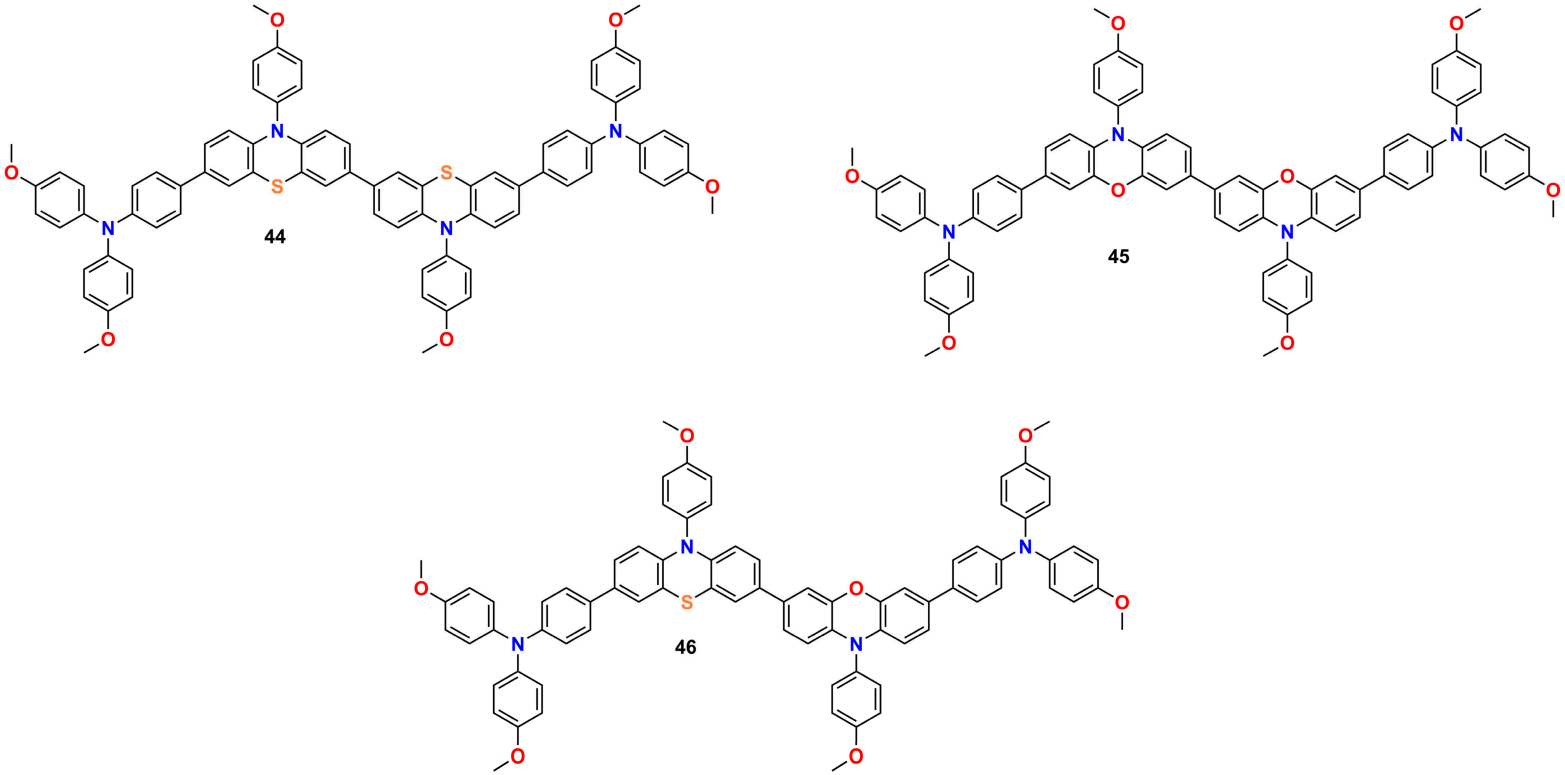
Chemical structure of phenothiazine and phenoxazine-based HTMs for PSCs.

Ding et al. [54] reported the synthesis of three new phenothiazine and phenoxazine-based HTMs **44**–**46** (Figure 10). The phenothiazine HTM **44** exhibits a high PCE of 23.09% in reverse configuration. But when the sulfur atom is replaced by an oxygen atom in the phenoxazine HTM **45**, the PCE decreases to 14.33% in the same configuration. The phenoxazine core results in a more planar molecular structure, which promotes π-π stacking and poor solubility during film formation. However, the phenothiazine core adopts a butterfly conformation, which significantly improves solubility and results in a more uniform film. Interestingly, combining them into a hybrid molecule **46** that synergistically combines the advantages of both systems results in an enhanced efficiency of 25.85% in the reverse configuration. All the HTMs exhibited high thermal stability, which is significantly higher than the phase-transition and thermal-decomposition temperatures of perovskite crystals. Additionally, the calculated costs of 44, 45, and 46 are 92.27 $/g, 105.97 $/g, and 94.30 $/g, respectively, which are much lower than spiro-OMeTAD 200 $/g.

**Figure 11:**
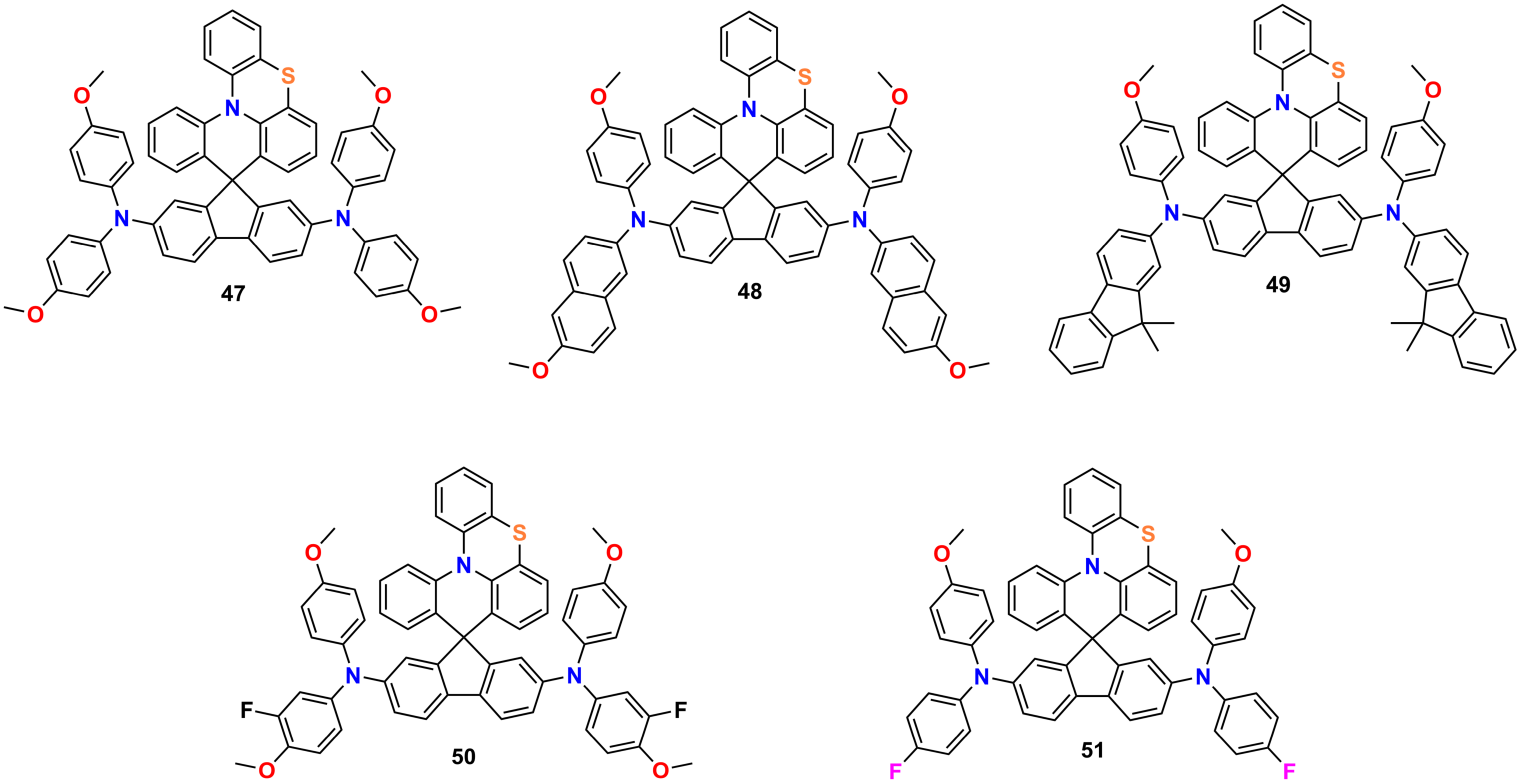
Phenothiazine–spiro-based HTMs for PSCs.

Mora and colleagues [18,55] synthesized different spiro derivatives based on phenothiazine (**47**–**51**, Figure 11) in three steps, finding that the five structures have absorption and emission properties that completely match those recorded for Spiro-OMeTAD. These compounds exhibited a narrow band around 300 nm, which was assigned to the optical characteristics of the peripheral groups, while a broad band around 350–400 nm was observed, derived from the π-conjugated structure of the central spiro nucleus. In the case of compounds **48** and **49**, the band showed a red shift due to the extended π conjugation between the peripheral naphthalene and fluorene units (Entries 27 and 28, Table 1). Furthermore, when estimating the optical band gap (*E_g_*), slightly higher values were observed for compounds **48**–**51** compared to spiro-OMeTAD (3.05 eV), demonstrating their potential as promising HTMs. Thermal analyses showed good stability with decomposition temperatures (T_dec_) of 400, 431, 420, 381, and 368 °C for **47**–**51**, respectively, with slightly higher T_dec_ values for asymmetric compounds containing fluorine atoms, such as **50** and **51**. The photovoltaic properties were studied in flat n-i-p devices using a standard ITO (**47**) or FTO (**48**–**51**)/SnO_2_/perovskite/HTM/Au configuration, highlighting the performance of compounds **48**–**51** in properties such as *V*_oc_, *J*_sc_, *FF*, and PCE, exceeding those obtained for Spiro-OMeTAD, achieving an efficiency of 25.75%, which was observed for compound **49** bearing a dimethylfluorene unit at the periphery. These compounds exhibited an excellent long-term stability studied in the dark at 25 °C, showing that all HTMs retained 95% of the initial PCE after 150 days (3600 h), with the exception of **51**, which showed a decrease in PCE of more than 30% in the same period of time, and under continuous irradiation of one unit of illumination, achieving a stability of 1000 h.

**Table 1:** Compilation of the optoelectronic and photophysical properties of compounds used in PSC ^[a]^.

Entry	Phenothiazine Derivatives	λ_max_ (nm)	HOMO (eV)	LUMO (eV)	*E_g_* (eV)	*V_oc_* (V)	*J_sc_* (mA·cm^−2^)	*FF* (%)	PCE (%)	Ref.
1	22 (encapsulated)	359	−5,01	−2,31	−2.70	0.95	14.32	72.86	10.83	[43]
2	23	447	−5.20	−2.66	−2.54	0.96	24.57	67.64	15.88	[44]
3	24	409	−4.97	−2.41	−2.56	1.01	19.75	49	9.77	[45]
4	25	412	−4.99	−2.45	−2.54	1.01	19.83	58	11.62	
5	26	430	−5.08	−2.49	2.59	1.11	25.1	69.8	19.4	[46]
6	27	473	−5.00	−2.58	2.42	1.165	25.54	77.3	23	
7	28	454	−5.05	−2.56	2.49	1.18	26.09	81.6	25.1	
8	29	437	−5.57	−2.73	2.84	1.126	23.65	78.83	20.99	[47]
9	30	393	−5.28	−2.12	3.16	0.99	19.69	72.3	13.9	[48]
10	31	383	−5.35	−2.11	3.24	1	21.08	74.18	15.8	
11	32	366	−5.38	−1.99	3.39	1.03	21.56	76.31	16.96	
12	33	305	−5.64	−2.55	3.09	1.041	23.36	66	16.05	[49]
13	34	309	−5.60	−4.80	2.85	1.047	23.79	72	17.93	
14	35	310	−5.71	−4.89	2.92	0.966	3.18	12.9	0.4	
15	36	335	−5.28	−2.42	2.86	1.11	23.7	75.5	19.9	[50]
16	37	330	−5.25	−2.46	2.79	0.98	19.9	67.6	13.2	
17	38	367	−5.25	−2.41	2.84	1.07	21.57	77.72	17.95	[51]
18	39	362	−5.21	−2.37	2.84	1.13	22.23	79.77	20.15	
19	40	363	−6.15	−2.85	3.03	1.10	11.43	33.77	4.24	[52]
20	41	319	−5.77	−2.52	3.25	1.18	23.36	80.02	22.06	
21	42	263.5	−5.33	−2.14	3.16	0.95	18.7	78.8	14.0	[53]
22	43	289.5	−5.22	−2.25	0.97	0.83	15.8	78.5	10.4	
23	44		−5.18	−2.26	2.92	1.13	24.98	81.82	23.09	
24	45		−5.10	−2.21	2.89	1.04	21.45	64.26	14.33	
25	46		−5.16	−2.21	2.50	1.19	25.75	84.36	25.85	
26	47	390	−5.14	−2.19	2.98	1.08	22.60	75	18.36	[55]
27	48	473	−5.20	−2.16	3.04	1.163	25.48	82.50	24.46	[18]
28	49	454	−5.19	−2.14	3.05	1.177	26.14	83.63	25.75	
29	50	437	−5.24	−2.12	3.14	1.173	25.69	83.67	25.22	
30	51	393	−5.28	−2.11	3.16	1.156	25.02	80.10	23.18	

^[a]^ Device parameters. *E_g_*: optical band gap is calculated according to *E_g_* = LUMO − HOMO. *V_oc_*: open-circuit voltage. *J_sc_*: short-circuit current densities. PCE: power conversion efficiency. The HOMO and LUMO values were calculated experimentally.

## 4. Dye-Sensitized Solar Cells (DSSC)

**Figure 12:**
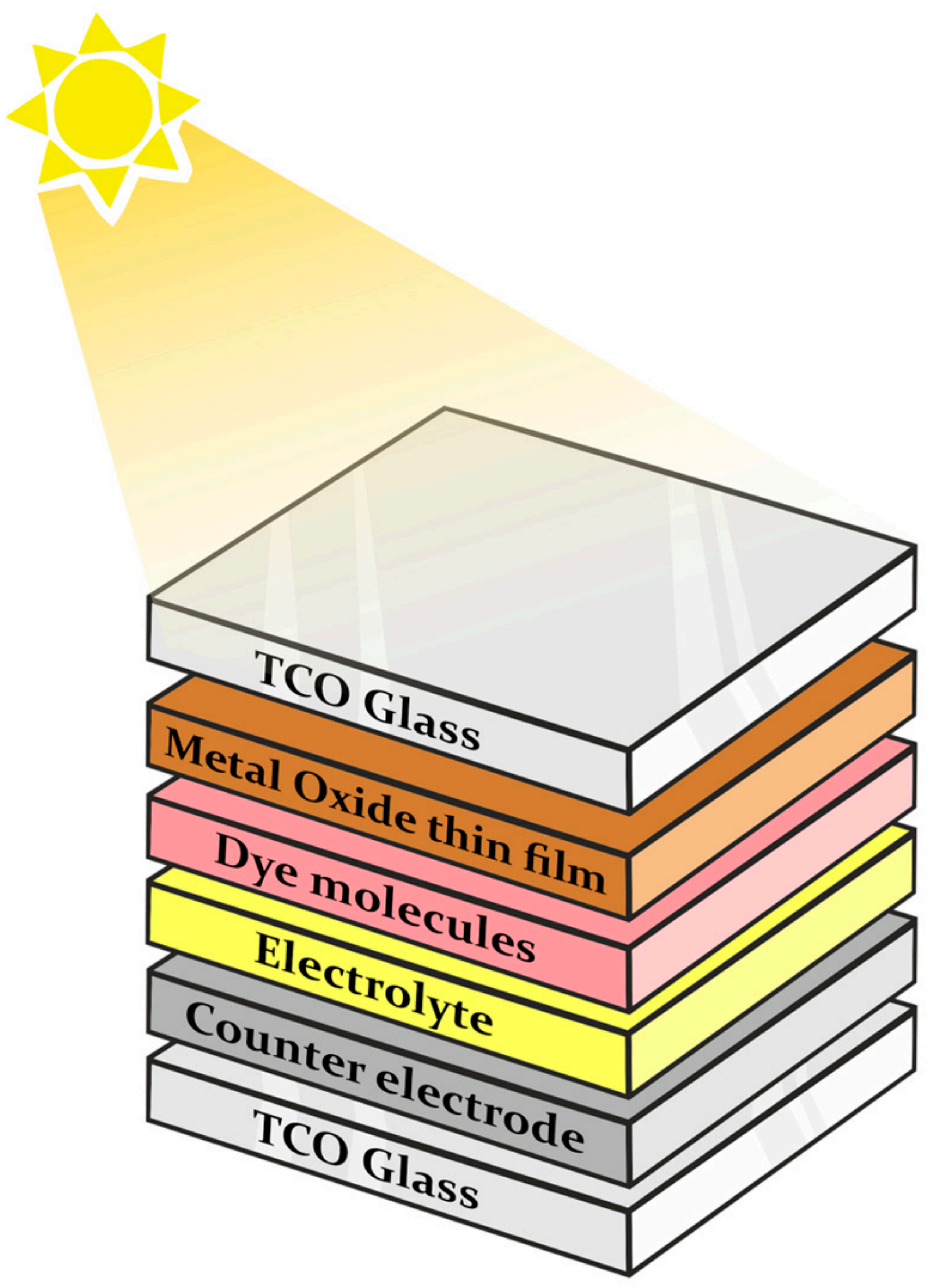
Architecture of DSSCs.

Dye-sensitized solar cells (DSSCs) are considered a promising solution due to their cost-effectiveness, ease of manufacture, low toxicity, and high energy conversion efficiency (PCE) [56]. These devices typically consist of (i) an electrical contact, a glass window coated with a thin transparent film of a conductive oxide (TCO), e.g., tin-doped indium oxide (ITO), (ii) a photoelectrode, a thin mesoporous film of a semiconductor, titanium oxide (TiO_2_) being the most common, (iii) a sensitizer, a monolayer of an organic, inorganic, or natural compound with a high molar absorptivity coefficient in the visible range, and (iv) a redox electrolyte, with only (3I^−^/I_3_^−^) or (Co^3+^/Co^2+^) pairs being commonly used. The last element is (v) a platinum counter-electrode coated with FTO (Figure 12) [57]. The dyes used in DSSCs must contain an anchoring group, which allows them to bind to the TiO_2_ semiconductor via a coordinated covalent bond.

**Figure 13:**
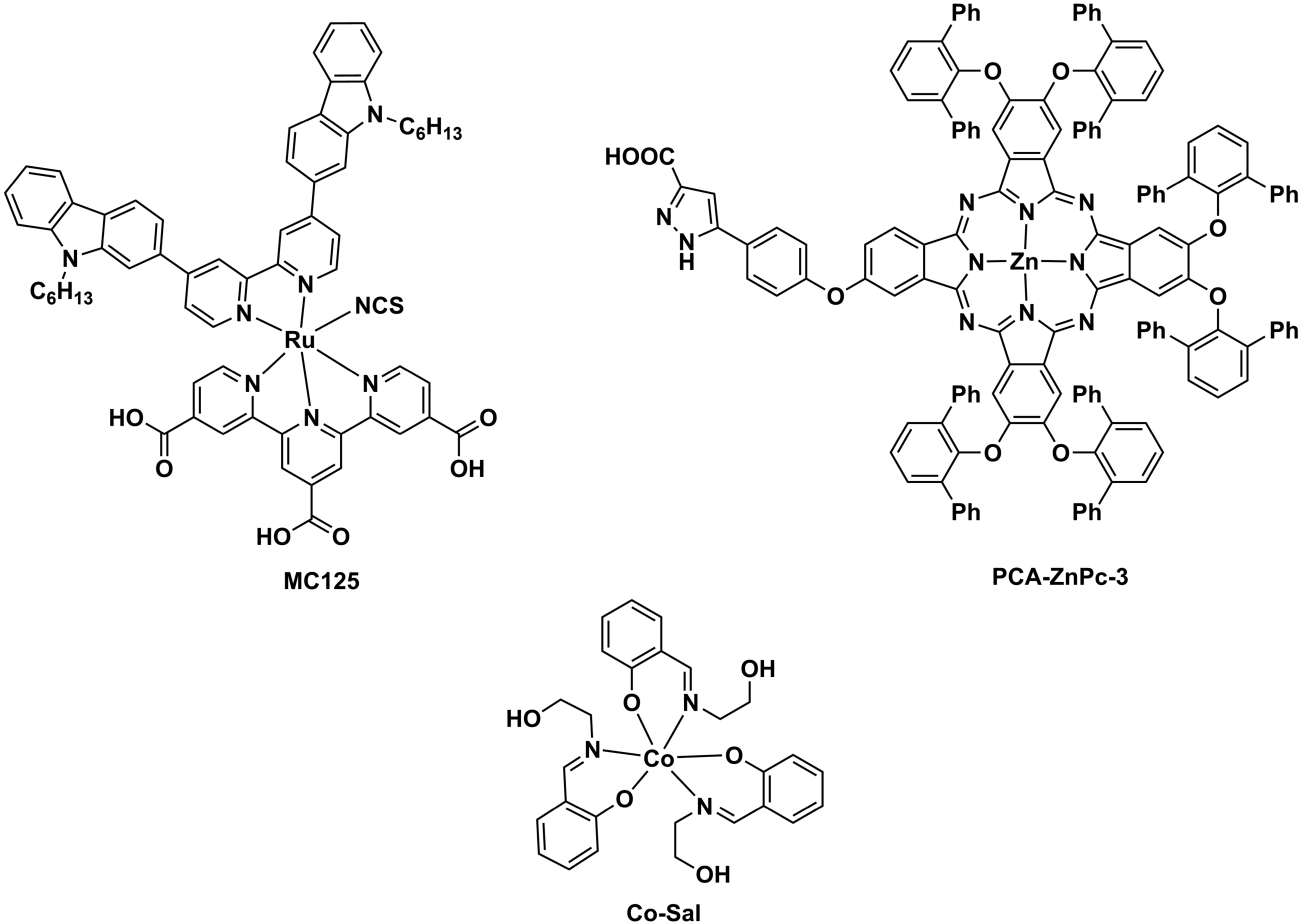
Chemical structure of some organometallic complexes for DSSCs.

The first generation of DSSCs used dyes derived from ruthenium complexes with polypyridine ligands, exhibiting good thermal stability, broad absorption spectra, and good electron transfer properties [57,58]. Over time, it was observed that using metals such as zinc, copper, iron, or cobalt, and ligands such as porphyrins, carbenes, or Schiff bases, improved cell performance, raising conversion efficiencies up to 13%. These compounds also showed broad absorption ranges in the visible spectrum and could be used as co-sensitizers, increasing the performance of cells sensitized with organic compounds (Figure 13) [59,60,61].

**Figure 14:**
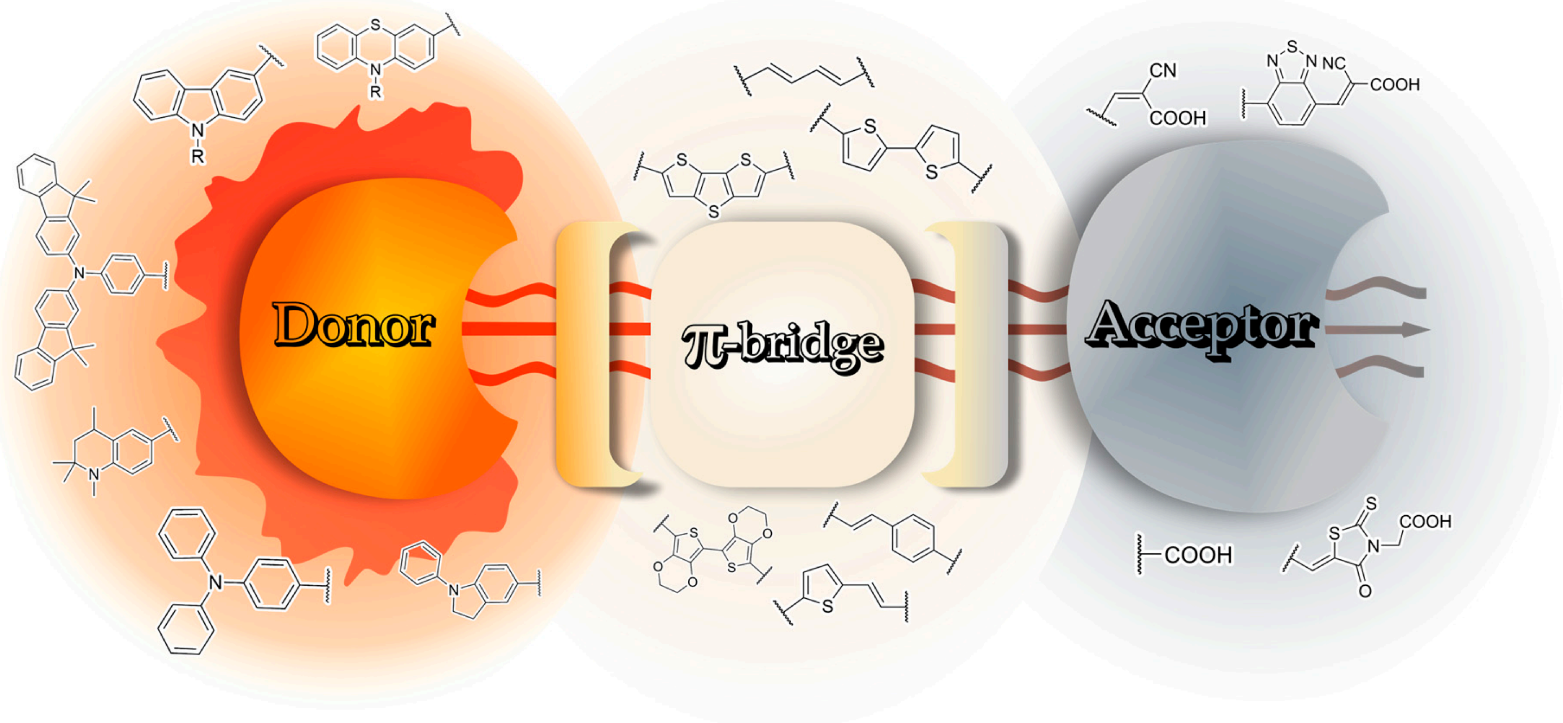
D-π-A dye structure and main groups used in the design.

In recent years, synthetic organic dyes have been the focus of several studies due to their advantages in design and synthesis, high absorptivity coefficients in the visible range, tunable band gaps, and lower production costs compared to organometallic complexes [62,63]. In the design of this type of dye, an acceptor–bridge–donor configuration is mainly used (A-B-D, A-π-D), where the donor group (with high electron densities) is attached to a bridge (group with extensive π conjugation), which in turn is attached to a charge-acceptor group that anchors it to the metal oxide of the DSSCs. Figure 14 shows some of the main groups used in the design of electroactive dyes, highlighting arylamines, phenothiazines, coumarins, carbazoles, thiophenes, and rhodanines [64,65,66,67,68].

### Phenothiazine in DSSCs

**Figure 15:**
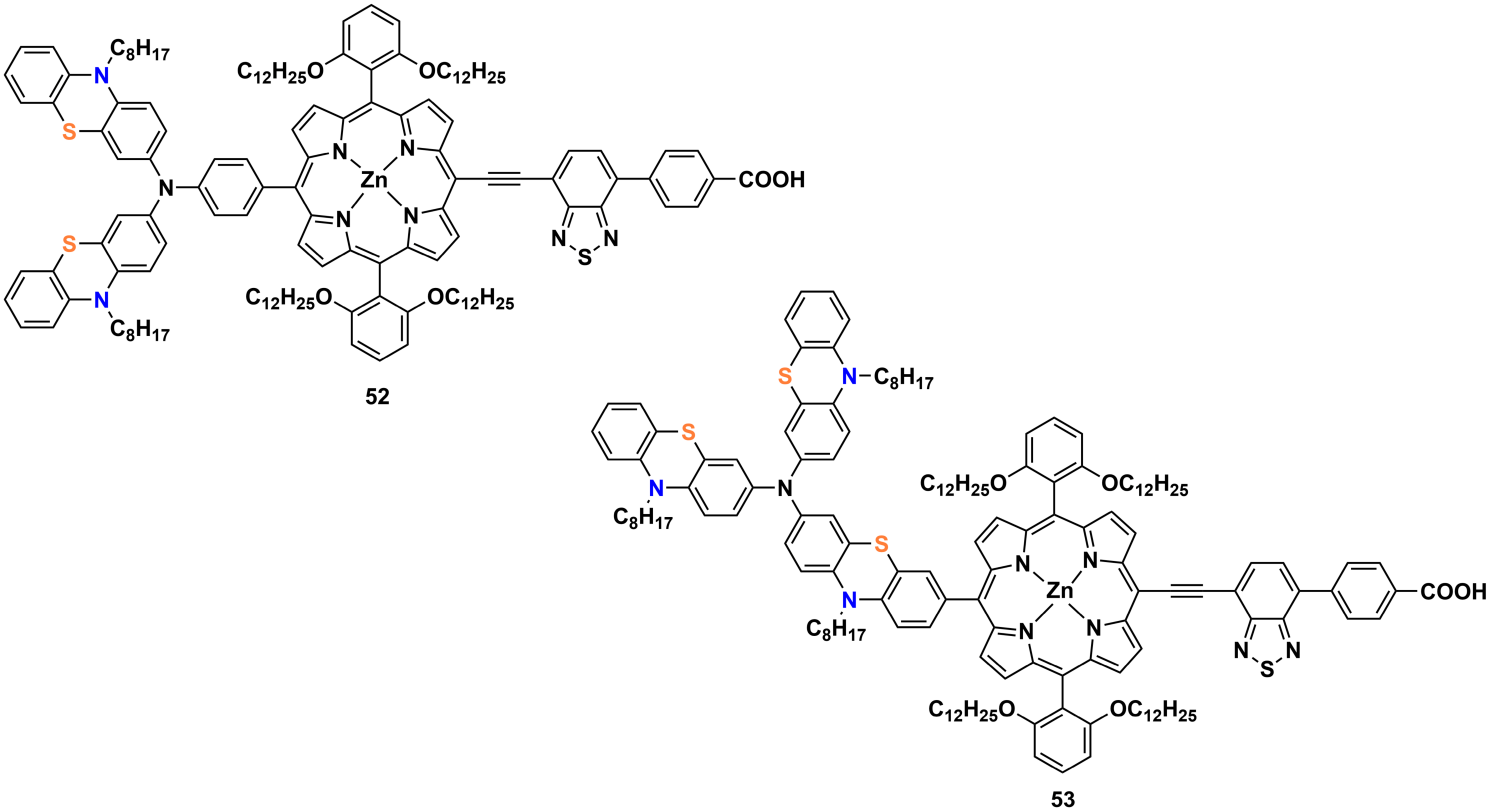
Phenothiazine–porphyrin-based dyes for DSSCs.

Li and collaborators designed two new phenothiazine–porphyrin-based dyes (**52** and **53**) for DSSCs applications (Figure 15) [69]. Porphyrin derivatives have also emerged as an attractive alternative for researchers due to their facile structural modification, exceptional redox potentials, and excellent absorption in the visible range. These dyes were achieved by introducing substitutions on the connecting donor unit (D) with multiple electron-rich moieties such as triphenylamine. The new D units were synthesized *via* nitration, nitro reduction, and C–N coupling reactions. The addition of the D unit to the zinc–porphyrin ring was carried out through Suzuki coupling. The optical property analysis revealed that both dyes exhibited two main maximum absorptions at 433 nm (ε = 115 × 10^3^ M^−1^ cm^−1^) and 643 nm (ε = 38.1 M^−1^ cm^−1^) for compound **52**, and 429 nm (ε = 76 M^−1^ cm^−1^) and 643 nm (ε = 15.7 M^−1^ cm^−1^) for compound **53** solutions. Both dyes exhibited an intense Soret band between 400 and 500 nm and a moderate Q band between 550 and 750 nm. The difference in molar extinction coefficients (ε) is associated with structural differences between these dyes, which give rise to intramolecular charge-transfer (ICT) processes. Furthermore, upon adsorption onto TiO_2_ films, the strong chelation between the carboxylic acid group and the TiO_2_ surface led to a broader absorption spectrum (400–700 nm) compared to the THF solution. This also resulted in higher dye loading on the films, with values of 2.09 × 10^−8^ mol cm^−2^ for compound **52** and 1.79 × 10^−8^ mol cm^−2^ for compound **53**. On the other hand, electrochemical studies showed that the solid-state oxidation potentials (Eₒₓ) for compounds **52** and **53** were 0.87 V and 0.82 V, respectively—both more positive than that of the I^−^/I_3_^−^ redox couple (0.4 V)—indicating sufficient driving force for dye regeneration. Additionally, the optoelectronic performance studies demonstrated that compound **52** exhibited a *V*_oc_ of 670 mV, *J*_sc_ of 16.84 mA·cm^−2^, *FF* of 70.02%, and PCE of 8.02%, superior to those obtained for compound **53**, which showed a *V*_oc_ of 650 mV, *J*_sc_ of 11.45 mA·cm^−2^, *FF* of 72.76%, and PCE of 5.41%. These results demonstrate the higher efficiency of compound **52** in DSSCs, which could be attributable to its largest loaded amount and corresponding highest ε that promote higher IPCE and *J*_sc_ values. Furthermore, the electron injection efficiency (η_inj_) and electron lifetime (τ) of device **52** were the highest. In addition, the theoretical calculation results also proved that the structure of compound **52,** containing two phenothiazines in the D unit, is more conducive to the ICT process. Thus, introducing a different number of phenothiazine units to replace the benzene units of triphenylamine in suitable positions is an advisable molecular engineering strategy.

**Figure 16:**
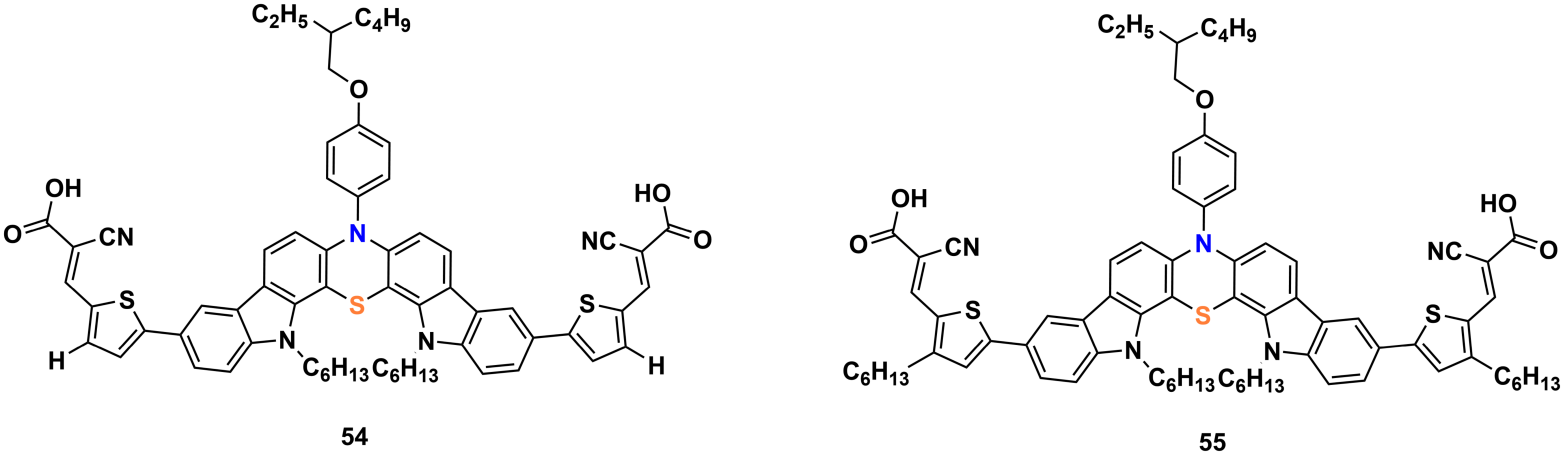
Phenothiazine–carbazole-based dyes for DSSCs.

Xia and colleagues synthesized two new phenothiazine–carbazole-based dyes **54** and **55** (Figure 16) [70]. The photophysical properties of the dyes were studied by UV-Vis absorption spectroscopy in a diluted THF solution (2 × 10^−5^ M), finding that both dyes exhibited typical dual absorption bands; the first one was located at shorter wavelength around 300–370 nm, arising from π-π* or n-π* transitions. The second absorption band in the visible region (370–550 nm) corresponds to ICT between donor and acceptor units. Compared to dye **54**, a shift towards the blue can be observed in dye **55**. This is due to the presence of the hexyl chain in the π-thiophen bridge, which distorts and reduces the planarity and conjugation of the dye’s molecular structure. The λ_max_ values for **54** and **55** were at 447 nm (ε = 64,100 M^−1^ cm^−1^) and 414 nm (ε = 57,600 M^−1^ cm^−1^), respectively. This indicates that both dyes have a high light-harvesting capacity due to their large π-conjugated structure. They also analyzed the normalized absorption spectrum of **54** and **55** on TiO_2_ films, finding that after anchoring, the resulting absorption spectrum (350–700 nm) in films was broader than in solution (300–550 nm), which benefits its use in capturing and converting sunlight. The electrochemical study showed that these dyes had similar ground-state oxidation potentials (E_ox_) of 1.30 V and 1.27 V for **54** and **55**, respectively, suggesting that the oxidized dyes can be efficiently regenerated. Moreover, the reduction potentials (E_red_) for both dyes were −1.24 V, which is more negative than the conduction band of TiO_2_ (−0.5 V), implying that the dyes have sufficient driving force to inject electrons into TiO_2_. The device’s performance study showed that **54** exhibited a considerable improvement in *J*_sc_, *V*_oc_, and PCE compared to **55**, with a higher *J*_sc_ for **54** (11.84 mA·cm^−2^) than in **55** (7.18 mA·cm^−2^). This was assigned to a higher molar extinction coefficient for **54** due to its better coplanarity and reduced steric hindrance for the absence of the hexyl chain, which results in a greater amount of dye adsorbed onto the TiO_2_. Likewise, compound **54** showed a PCE value of 5.97%, whereas compound **55** showed a PCE value of 4.28%. To improve the photovoltaic properties of the cells, the dyes were co-sensitized with WL8. Compound **55** exhibited a molar extinction coefficient in the range of 350–450 nm in the UV-Vis spectrum, while WL8 exhibits a high molar extinction coefficient in the 450–550 nm range, indicating that they complement each other relatively well. This significantly improves the optoelectronic properties with a *J*_sc_ of 13.67 mA·cm^−2^, *V*_oc_ of 0.70 V, and a PCE of 6.69%, which is significantly higher than the individual dyes **55** and WL8, and is approximately 90% of the PCE of N719 under the same conditions.

**Figure 17:**
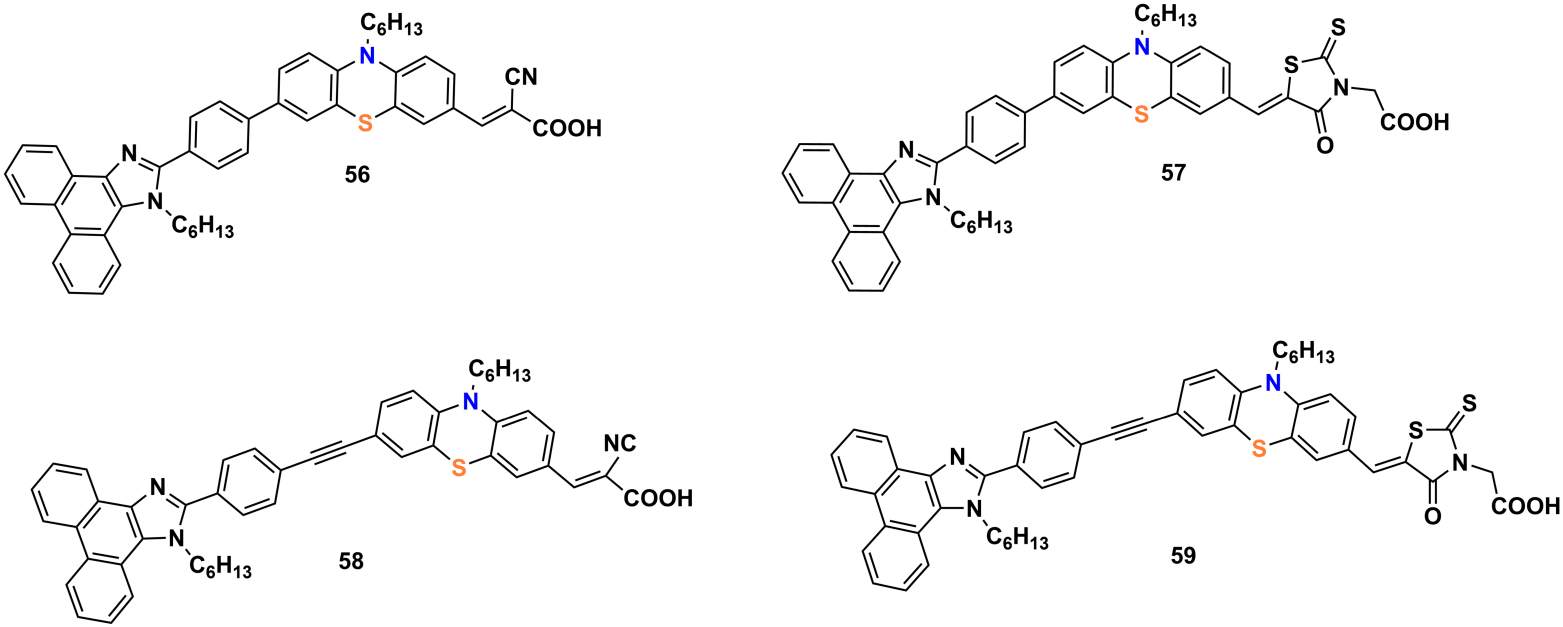
Phenothiazine–phenanthroimidazole-based sensitizers for DSSCs.

Duvva et al. synthesized four new sensitizers (**56**–**59**) with a D-π-A architecture, using substituted phenanthroimidazole and phenothiazine as donors and rhodanine 3-acetic acid and cyanoacetic acid as electron acceptors and anchoring units for TiO_2_ (Figure 17) [71]. These compounds were prepared by synthesizing the key intermediates *via* a Sonogashira coupling, followed by a Knoevenagel condensation with rhodanine 3-acetic acid or cyanoacetic acid. Analysis of the absorption and emission properties of these compounds revealed that all of them presented three or four absorption bands between 220 and 600 nm, attributing the bands between 220 and 400 nm to the π-π* transitions corresponding to the donor moieties of the dyes; the bands around 400–650 nm were attributed to ICT processes from the donor region to the acceptor fraction. The maximum absorptions were observed at 259 and 260 nm for compounds **56** and **57**, respectively. While compounds **58** and **59** showed a red shift with maximum absorption bands at 259 and 259 nm, the red shift in compounds **57** and **59** compared to their counterparts (**56** and **58**) was attributed to the strong electron-attracting character of rhodanine 3-acetic acid; however, the addition of the ethynyl group improves electron injection into the conduction band of TiO_2_. The performances of these dyes in solar devices showed a low dye load on the surface of TiO_2_ of the cells for compounds **58** and **59** compared to **56** and **57**; this was attributed to the larger molecular size of dyes **58** and **59**, compared to **56** and **57**, due to the presence of the ethynyl group; the photovoltaic yields of the devices were characterized at 100 mW/cm^2^ under simulated sunlight AM 1.5 G, finding that the dyes **56** and **58** exhibited a *J*_sc_ of approximately 16.70 mA·cm^−2^; however, dyes **57** and **59** showed a *J*_sc_ of approximately 14 mA·cm^−2^. The *V_oc_* of the four dyes ranged from 0.75 V to 0.80 V, and the *FF* was between 0.68 and 0.71. The PCE values for **56** and **58** were 8.80% and 9.05%, while those for **57** and **59** were only 7.83% and 7.90%, respectively. This was reflected in the IPCE spectra, where **58** and **59** were broader and extended to 780 nm with a maximum absorption of approximately 75% around 500 nm, while for **56** and **57**, the maximum absorption was at 480 nm, extending to 650 nm. The above was attributed to the relatively short lifetime of electrons in the excited state and the isolation of the LUMO orbital from the anchor groups, resulting in low values of *J_sc_*, low injection of electrons into the conduction band of TiO_2_, and rapid recombination of charges, leading to low *V_oc_*.

**Figure 18:**
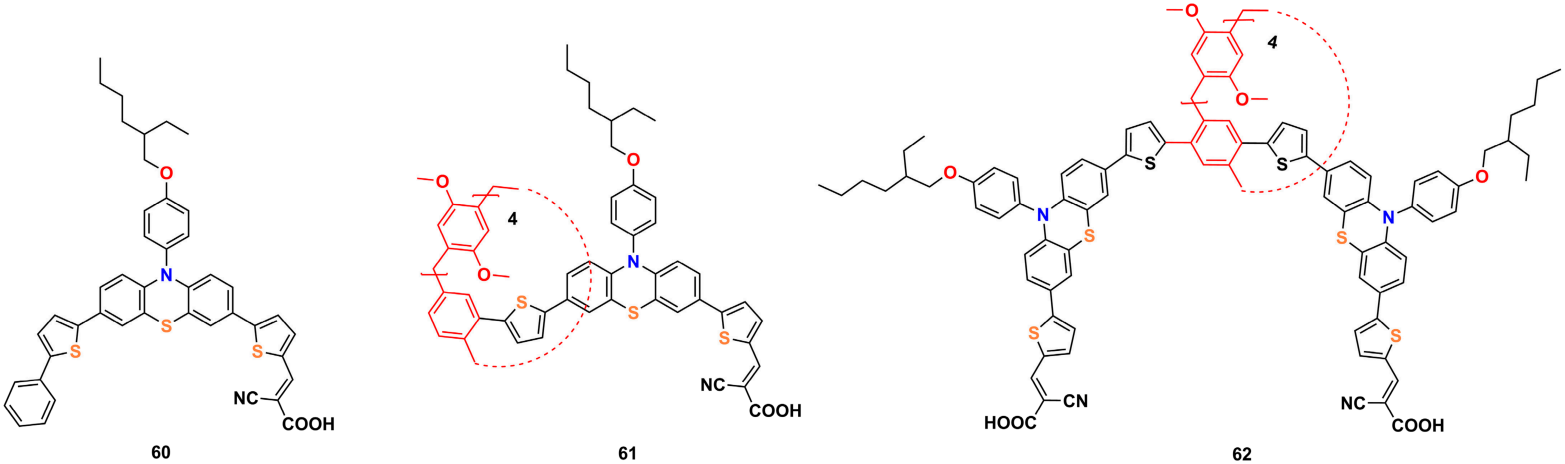
Phenothiazine–pillar[5]arene-based sensitizers for DSSCs.

Liao and coworkers conducted a study of dyes (**60**–**62**) derived from pillar[5]arene and phenothiazine (Figure 18) [72], observing that compound **60** exhibited a maximum absorption wavelength at 422 nm, which is related to π-π* transitions and ICT, while compounds **61** and **62** showed a red shift with wavelengths at 481 and 488 nm, respectively, related to energy transfer between the pillar[5]arene and the main chain unit. They also observed shifts in λ_max_ when the medium was acidified, with values of 448, 504, and 504 nm for compounds **60**, **61**, and **62**, respectively, while in an alkaline medium, the shift was observed toward 408, 446, and 451 nm, respectively. These results are due to the hypsochromic shift caused by the low intensity of the ICT resulting from the deprotonation of the carboxylic acid group. The HOMO level of these dyes remained constant at 0.91, 0.87 eV, and 0.86 V for **60**, **61,** and **62**, respectively, due to the similar structure of the skeleton (D-π-A) of dyes. Meanwhile, the LUMO levels remained between −1.55 and −1.22 eV. Therefore, the dyes have energy levels well-suited to TiO_2_ and electrolytes in DSSCs. Furthermore, the study of their photovoltaic efficiencies showed that the device based on dye **62** had a PCE of 8.36% with a *V*_oc_ of 746 mV and *J*_sc_ of 15.63 mA·cm^−2^, higher than the 7.12% of the device designed with **61,** which showed optoelectronic parameters relatively close to those of **62** with a *V*_oc_ of 674 mV and *J*_sc_ of 14.66 mA·cm^−2^. This was assigned due to the introduction of the pillar[5]arene moiety, which can suppress dye aggregation and charge recombination, leading to an improved *V*_oc_ by 42–73 mV (6–10%). Regarding the *J*_sc_ for the three dyes, it was observed that the pillar[5]arene-phenothiazine conjugation in **61** and **62** showed different trends compared to the unconjugated dye **60**. Analysis of the short-circuit current density (*J_sc_*) for the three dyes revealed that the pillar[5]arene–phenothiazine derivatives **61** and **62** exhibited trends different from that of the one lacking this fragment (**60**). To study this, they measured the number of dyes anchored to TiO_2_, finding that compound **60** had the highest dye load (2.43 × 10^−7^ mol·cm^−2^), exceeding **61** (1.92 × 10^−7^ mol·cm^−2^) by 26% and **62** (1.77 × 10^−7^ mol·cm^−2^) by 37%.

**Figure 19:**
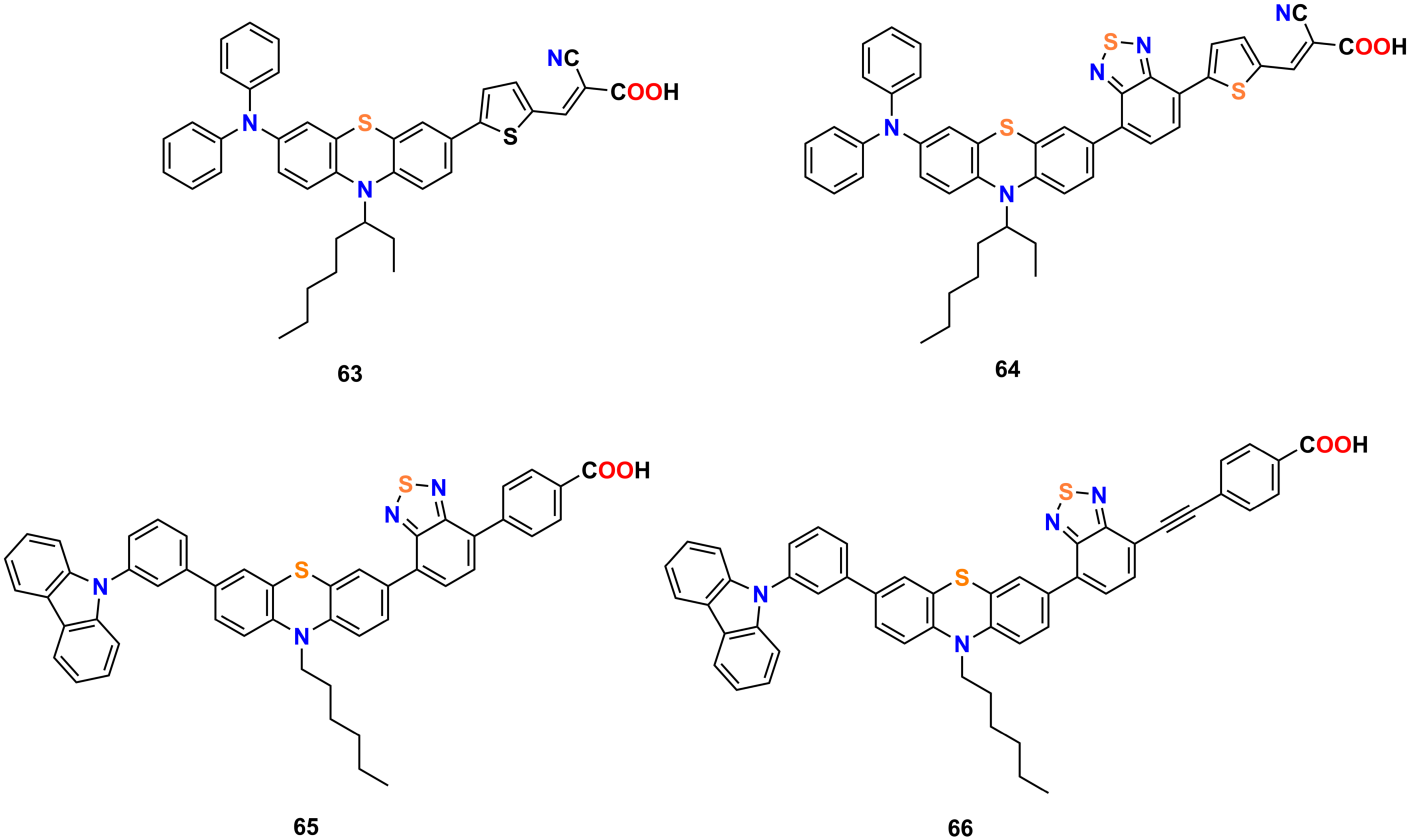
Dyes with D–π–A and D–A–π–A architectures used in DSSCs.

Li and colleagues synthesized two new phenothiazine-based organic dyes with D–π–A (**63**) and D–A–π–A (**64**) architectures (Figure 19) [73]. The studies of their photophysical and electrochemical properties showed that the addition of an auxiliary acceptor in **61** caused a red-shift of 57 nm in comparison to **63**, with a λ_max_ absorption of 449 nm and ε of 62.3 × 10^3^ M^−1^ cm^−1^ for **63** and 506 nm with ε of 62.3 × 10^3^ M^−1^ cm^−1^ for **64** in THF solutions. Likewise, the oxidation-reduction potential was determined by cyclic voltammetry, obtaining potentials of −2.27 and −2.07 V for **63** and **64**, respectively. Additionally, Li and colleagues performed theoretical calculations and found that the HOMO of the dyes was mainly located in the donor units, while the LUMO is distributed in the fractions π-A o A-π-A. Finally, photovoltaic yields show that the IPCE of the cell **63** was greater than **64** in the range of 300–560 nm, and the maximum IPCE value for **64** was 58%, which is significantly lower than that of the cell made with **63** (78%); this is probably due to the higher molar extinction coefficient of **63** with respect to that of **64** in this range. However, the IPCE of the cell made with **64** showed a broader spectral response than the cell with **63**, with a maximum range close to 800 nm, which is probably due to the presence of the benzothiazole unit. In addition, the results revealed that the *V*_oc_ for **63** (710 mV) is significantly larger than that for **64** (640 mV). Finally, the PCE for **63** was 5.35%, which is greater than the PCE of the cell made with **64,** with a PCE of 4.43%, consistent with the IPCE analysis; it may also be due to the high molar extension coefficient for **64**, as well as the short half-life of the device’s electrons. Likewise, Ding’s team synthesized new dyes (**65** and **66**, Figure 19) with acceptor moieties based on benzothiadiazole (BTD) and benzoic acid (BA) (**65**, BTD-DA) and 4-(benzo[c][1,2,5]thiadiazol-4-ylethynyl)benzoic acid (**66**, BTD-EBA), and donor fragments based on carbazole–phenothiazine units. The photophysical and electrochemical results showed that in DCM solution, both dyes exhibited three absorption peaks between 280 nm and 470 nm, attributed to π-π* transitions and ICT processes. They also observed a bathochromic shift of 21 nm for **66** compared to **65**, since **66** exhibits stronger intramolecular charge-transfer processes because of the flatter molecular structure resulting from the addition of the ethynyl group between the acceptor and the anchoring group, which increases the ε by about 2000 units. However, when the dyes were adsorbed onto TiO_2_, a red shift was observed for the ICT process, indicating the formation of *J*-aggregation. Furthermore, the HOMO levels obtained were the same for both dyes (−5.57 eV) while the LUMO levels were −3.43 eV and −3.55 eV for **65** and **66**, respectively. This decrease was attributed to the ethynyl group between the aromatic rings, which reduces the torsion angle and promotes delocalization of the frontier molecular orbitals. The photovoltaic results showed that **65** exhibited a PCE of 6.69% with a *J_sc_* of 13.33 mA·cm^−2^ and a *V_oc_* of 0.81 V, while **66** exhibited a high *J_sc_* value of 14.04 mA·cm^−2^ and a similar *V_oc_* of 0.80 V, leading to a PCE improvement of 7.33% [74].

**Scheme 9:**
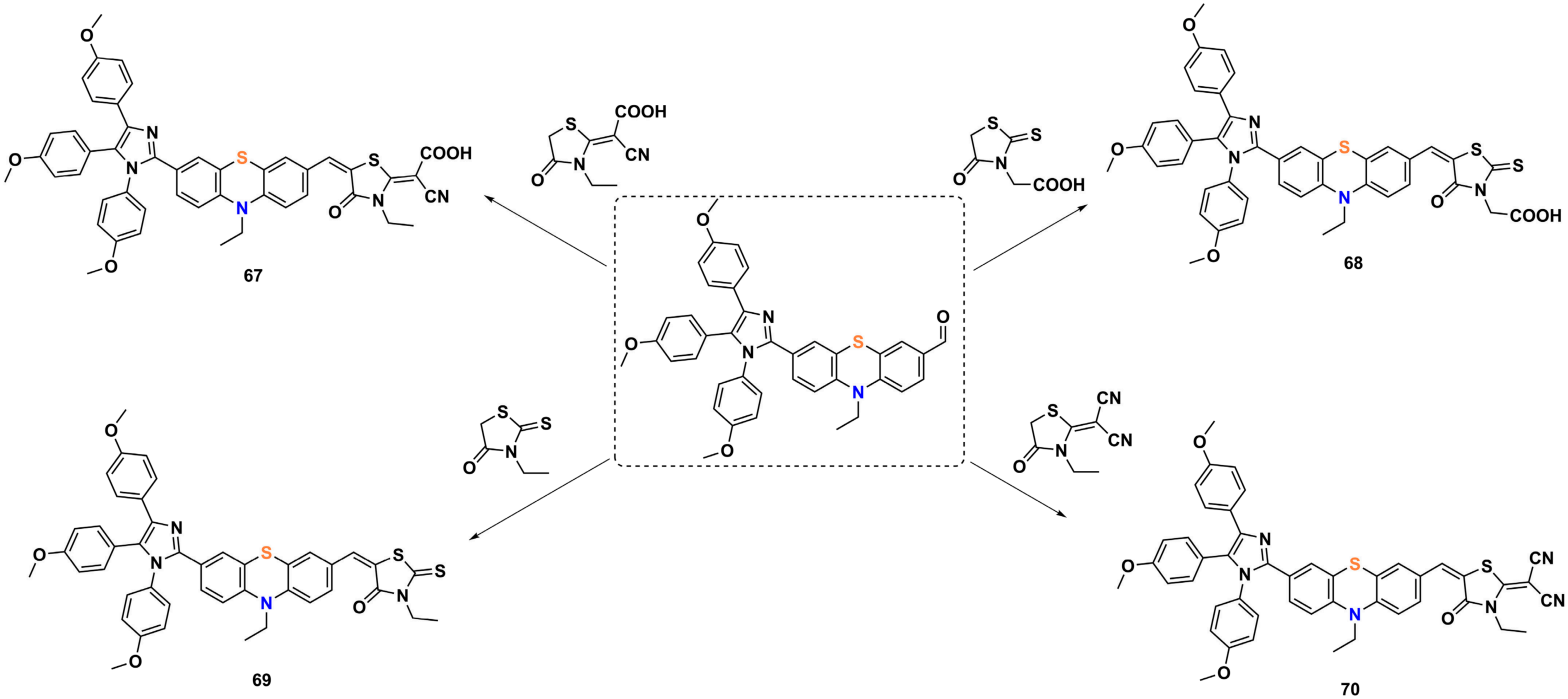
Structures of new dyes with a D–D′–A (imidazole–phenothiazine–rhodanine) architectures for DSSCs.

The Syiemlieh group explored various molecular architectures of new dyes with donor–auxiliary donor–acceptor structures (**67**–**70**, Scheme 9) [75]. These dyes featured *N*-aryl-substituted imidazole as the primary donor unit, a *N*-alkylated phenothiazine as the auxiliary donor, and different rhodanine derivatives as the acceptor. UV–Vis absorption and fluorescence studies in toluene revealed two characteristic absorption bands for all dyes between 250 and 600 nm. The higher-energy band was assigned to π–π* transitions, while the broad band spanning 380–600 nm corresponded to the ICT band between the donor and acceptor units. A hypsochromic shift was observed upon the addition of triethylamine (TEA) or trifluoroacetic acid (TFA), reflecting changes in donor–acceptor interactions due to protonation/deprotonation of the cyanoacrylic acid moiety. Fluorescence spectra showed that all dyes emit at long wavelengths, attributed to the strong electron-withdrawing character of the anchoring groups, which enhances the ICT nature of the dyes. Computational studies yielded HOMO levels of −4.89 eV for **67** and **68**, −4.84 eV for **69**, and −4.97 eV for **70**, and LUMO energies of −2.54, −2.56, −2.42, and −2.68 eV for **67**–**70**, respectively. The photovoltaic characterization revealed that dye **67** delivered the best performance, with a *J*_sc_ of 13.08 mA·cm^−2^, *V*_oc_ of 0.79 V, and a PCE of 6.96%, closely matching the values of the benchmark N719 dye (*J*_sc_ 12.36 mA·cm^−2^, *V_oc_* 0.80 V, PCE 6.97%). This was attributed to an efficient electron injection into TiO_2_ through the cyanoacetic group, while compound **68**, bearing rhodamine-3-acetic acid, relying on a through-space charge transfer, experienced a significant recombination loss, affording a much lower PCE value of only 0.26%. All dyes exhibited multistep thermal degradation, with significant weight losses between 250 and 600 °C, confirming their suitability for DSSC applications.

**Figure 20:**
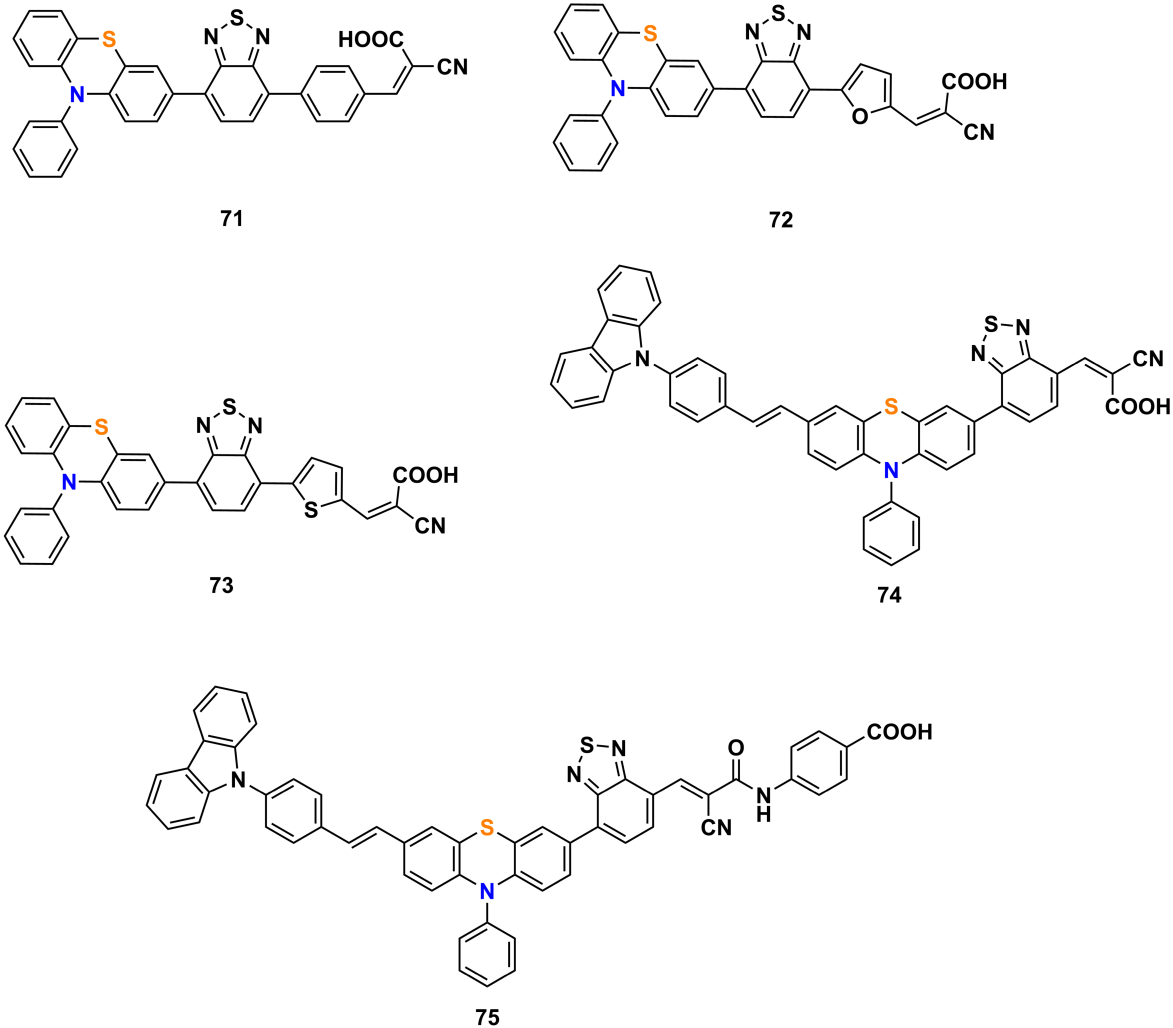
Benzothiadiazole- and phenothiazine-based dyes used in DSSCs.

Several research groups have investigated benzothiadiazole as an electron-accepting unit in dye molecules. Han and colleagues [76] synthesized three new dyes (**71**–**73**) with a D-A-π-A structure using benzothiadiazole as an auxiliary acceptor, phenothiazine as a donor unit, aryl ring as π bridges, and cyanoacetic acid as the main acceptor and anchoring unit (Figure 20). They found that these dyes exhibited three absorption peaks due to the addition of the benzothiazole moiety, where the peak between 300 and 350 corresponds to π-π* transitions, while the peaks at 450–500 nm are caused by intramolecular charge transfer from the *N*-phenylphenothiazine donor to the cyanoacetic acid acceptor. The benzothiadiazole bridge generated bands between 400 and 450 nm for **72** and **73**, but for **71**, the band shifted to 350–400 nm. Although the dyes were structurally similar, the different π bridges caused changes in the absorption spectra, where compound **73** shows better absorption capacity in the visible region, with an absorptivity coefficient almost double that of compounds **71** and **72**, while when using a furan bridge, the absorption spectrum shows a notable decrease and shift towards red compared to thiophene; moreover, when using benzene as a bridge, there is a shift towards blue on the part of the ICT band. Furthermore, the LUMO energy levels of 68–70 are between −3.15 and −3.05 eV, which are higher than the TiO_2_ conduction band (−4.0 eV), suggesting that the dyes can effectively inject electrons into the TiO_2_ film upon light absorption. Meanwhile, the HOMO energy levels were found to be in the range of −5.48 to −5.43 eV, which are lower than the redox potential of the I_3_^−^/I^−^ electrolyte (−4.9 eV), indicating that the dyes’ oxides can accept electrons from the electrolyte and successfully regenerate, to complete the cell circuit. For their part, the optoelectronic parameters of the devices designed with the dyes showed high *V*_oc_ values of 0.69 eV, *J*_sc_ of 14.25 mA·cm^−2^, and PCE of 6.76% for dye **71**, while for compounds **72** and **73**, the parameters remained relatively close, with *V*_oc_ between 0.62 and 0.59 eV, *J*_sc_ of 11.16 and 8.76 mA·cm^−2^, and PCE of 4.84 and 3.94%. On the other hand, Elmorsy et al. synthesized new sensitizers with a D-D-π-A structure based on phenothiazine and carbazole, using Wittig reactions between the carbazole fraction and Suzuki couplings with benzothiadiazole, using cyanoacetic acid (**74**) and 4-(2-cyanoacetamido)benzoic acid (**75**) as anchoring groups. The study of their electrochemical and photophysical properties showed strong absorption in the visible range (400–600 nm), which can be attributed to ICT between the electron-rich groups (phenothiazine and carbazole) and the electron-accepting groups. In turn, compound **75** presented a red-shifted absorption maximum compared to **74**, attributed to its extended conjugation and greater delocalization of its electrons due to the addition of 4-(2-cyanoacetamido)benzoic acid. Computational calculations showed that the HOMO energy levels showed a difference of 0.30 eV between **74** and **75**, with values of −5.62 and −5.32 eV, respectively, while the LUMO electronic level values were −3.58 and −3.40 eV for **74** and **75**, respectively, which suggests that oxidized sensitizers can be regenerated by the electrolyte after injecting electrons into the conduction band of TiO_2_. Meanwhile, the photovoltaic parameters revealed that both dyes presented higher values in *V*_oc_ (0.825 and 0.866 V), *J*_sc_ (18.96 and 19.98 mA·cm^−2^), *FF* (65.27 and 67.61%), and PCE (10.21% and 11.70%) compared to N719 (*V*_oc_ = 0.774 V, *J*_sc_ = 17.01 mA·cm^−2^, *FF* = 57.72%, and PCE of 7.60%). Additionally, the device designed with the N718 (co-sensitizer) (top) and **72** (bottom) structure in P-Tamdem showed a significant improvement in the parameters of *V*_oc_ (0.9 V), *J*_sc_ (21.01 mA·cm^−2^), *FF* (67.95%), and PCE (12.85%) [19].

**Figure 21:**
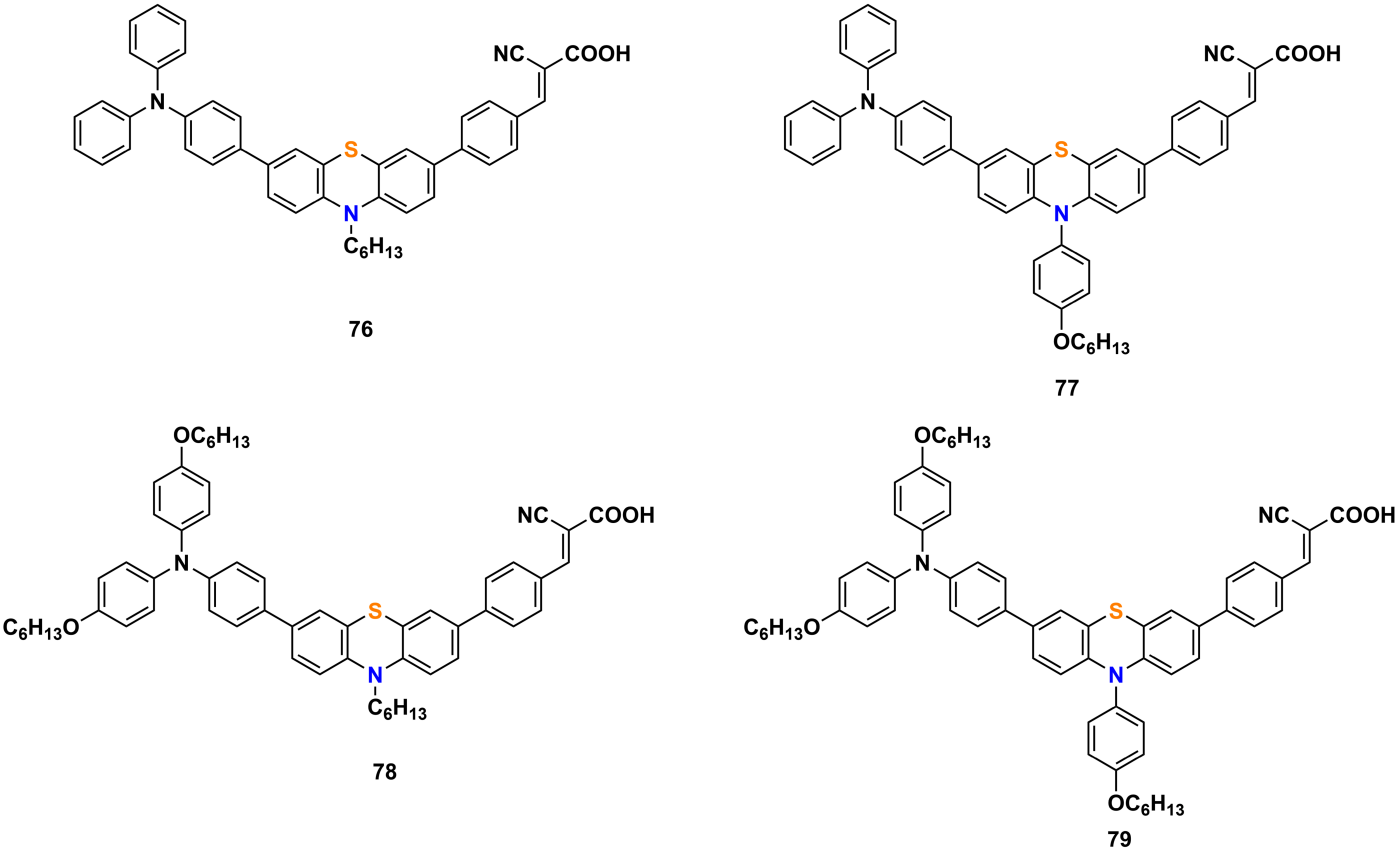
Dyes based on triphenylamine and phenothiazine with D-D-π-A architectures for DSSCs.

To analyze the impact of alkyl and alkoxy substituents, Xu and colleagues synthesized new dyes with a D-D-π-A structure (**76**–**79**, Figure 21) [77] using a phenyl group as a π-bridge. When studying the light absorption capacity of the dyes, they found that due to their similar structure, the four dyes exhibit similar absorption spectra and molar extinction coefficients (**76**: λ^π-π*^ 330 nm (ε 48.944 M^−1^ cm^−1^), λ^ICT^ 433 nm (ε 14,839 M^−1^ cm^−1^); **77**: λ^π-π*^ 336 nm (ε 48,573 M^−1^ cm^−1^), λ^ICT^ 461 nm (ε 15,273 M^−1^ cm^−1^); **78**: λ^π-π*^ 334 nm (ε 48,995 M^−1^ cm^−1^), λ^ICT^ 443 nm (ε 14,909 M^−1^ cm^−1^); **79**: λ^π-π*^ 337 nm (ε 49,567 M^−1^ cm^−1^), λ^ICT^ 464 nm (ε 14,871 M^−1^ cm^−1^)) with two main absorption bands around 330 nm and at 450 nm assigned to π–π* and ICT bands, respectively. The band in the region of 300 to 400 nm corresponds mainly to π-π* transitions from the aromatic skeleton of the peripheral groups to phenothiazine, while the less energetic transitions can be attributed to ICT processes from the donor to the acceptor. Furthermore, when comparing **76** without alkoxy modification, the π-π* absorption peak of **77**, **78**, and **79** exhibited a red shift of 6, 4, and 7 nm, respectively, while the band associated with ICT had a shift of 28, 10, and 31 nm, respectively, which indicates that the addition of alkoxy groups to the donor or auxiliary donor can result in a red shift in absorption. The incorporation of alkoxy phenyl groups at the *N*-position of phenothiazine in compounds **77** and **79** generates red shifts in ICT processes, attributed to a greater electron-donating capacity of the alkoxy phenyl group, and improves both the electronic transitions and the ICT processes of the dyes. The absorption of each dye in TiO_2_ showed an absorption band around 400 nm associated with ICT character. This shift towards blue can be attributed to the formation of *H*-aggregates or the deprotonation of carboxylic acid after adsorption on the TiO_2_ surface. The electrochemical study showed that the four dyes exhibit two reversible oxidation processes, indicating good redox stability and good dye regeneration in DSSCs. The two redox waves are attributed to the oxidation of the triphenylamine fraction and the phenothiazine segment. In turn, the HOMO energy levels—found to be **76** (−5.16 eV), **77** (−5.14 eV), **78** (−5.11 eV), and **79** (−5.09 eV)—revealed increases attributed to the improvement in the ability of the dyes to donate electrons after the modification of the alkoxy groups, while the LUMO energy levels remained constant at around −2.87 and −2.88 eV. These values were approximately 1.1 eV higher than the conduction band of TiO_2_, ensuring an effective electron injection from the dye to TiO_2_. Moreover, since the HOMO energy level is 0.35 eV deeper than the I^−^/I_3_^−^ redox pair, rapid dye regeneration is guaranteed. For their part, photovoltaic yields showed that **76** had a PCE of 5.38% with a *J*_sc_ of 11.03 mA·cm^−2^, *V*_oc_ of 0.84 V, and *FF* of 58%. The device manufactured with **77**, which incorporates an alkoxy phenyl group at the *N* position of the phenothiazine and lacks of alkoxy in the triphenylamine moiety, mitigated charge recombination and exhibited the strongest and widest IPCE spectrum, the highest *J*_sc_ (12.77 mA·cm^−2^), *V*_oc_ (0.85 V), and *FF* (60%) values leading to the highest PCE (6.59%) among these derivatives. The addition of two alkoxy groups to the triphenylamine unit results in decreases in PCE, *J*_sc_, *V*_oc_, and *FF* for **78** and **79**.

**Figure 22:**
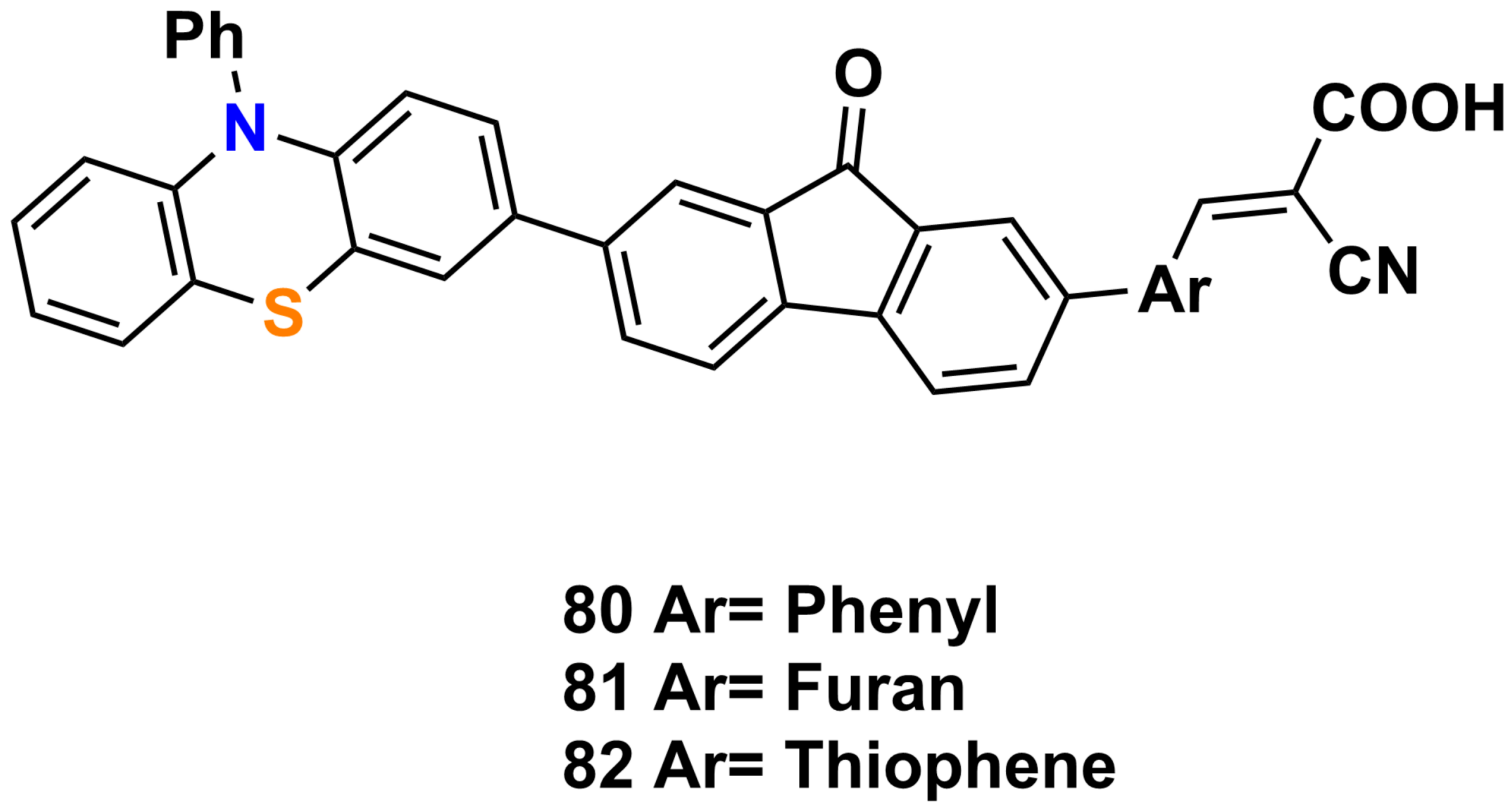
Phenothiazine–fluorenone-based dyes for DSSCs.

Han’s group designed new dyes with a central fluorenone unit, exploring the use of different π-bridges (phenyl **80**, thiophene **81**, and furan **82**, Figure 22) [78], where the results of the optical properties showed that the three dyes exhibited two absorption bands: one between 275 and 290 nm, attributed to high-energy π-π* transitions, and the second between 350 and 420 nm, caused by ICT transitions from the phenothiazine electron donor to the cyanoacrylic acid acceptor through the fluorenone unit and different aromatic ring bridges. Furthermore, it was found that the different aromatic bridges change the absorption of the ICT transitions, with red shifts observed for **81** and **82**, exhibiting bands around 417 and 408 nm, respectively. This may be due to the improved flatness of the dyes derived from thiophene and furan bridges, which leads to a higher conjugation system and promotes ICT transitions; while the phenyl ring, being more voluminous and possessing greater resonance energy, induces less effective conjugation for **80**, which generates a shift to blue for the ICT transition at 365 nm. When the dyes were added to TiO_2_, a blue shift was observed in the ICT transitions due to the formation of COO-Ti^2+^ caused by the lower withdrawal of the carboxyl anion relative to the carboxylic acid, inducing a blue shift due to the face-to-face alignment (H-aggregation) of the dye in TiO_2_. These dyes are also stacked in head-to-tail mode (*J*-aggregates), which generates red shifts in the ICT transitions. The IPCE in the range of 350–500 nm was found to be 61% at 406 nm for **82**, while **80** and **81** had maximum IPCE values of 57% at 490 nm and 56% at 500 nm, respectively. The *V*_oc_ of these dyes was in the range of 0.64 to 0.72 V in the order **80** > **81** > **82**, due to the electron injection efficiency and the charge recombination ratio. In contrast, *J*_sc_ remained in the range of 11.46 to 12.88 mA·cm^−2^, with dyes derived from small rings such as thiophene and furan exhibiting better light-absorption ranges and planarity, reflected in higher *J_sc_* and *FF*. On the other hand, the PCEs obtained were in the range of 5.24 to 5.75%, with **81** standing out with a PCE of 5.75% with a furan bridge.

**Figure 23:**
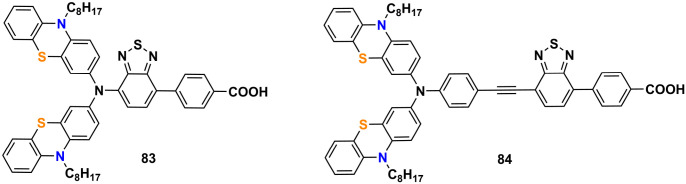
Phenothiazine-triphenylamine-based organic dyes for DSSCs.

Huang and colleagues explored the effect of conjugated groups for favorable molecular planarity and suppression of charge recombination in systems based on double phenothiazine units directly linked to a triphenylamine core endowed with benzothiadiazole moieties as auxiliary acceptors and benzoic acid acceptor and anchoring unit (**83** and **84**, Figure 23) [79]. The photophysical and electrochemical properties showed absorption bands within the visible range caused by ICT processes from the electron donor unit (2-phenothiazine-amine) to the acceptor fraction (benzoic acid) in the molecules, finding the spectral parameters contained in Table 2 (Entries 1 and 2). Compound **84** showed a blue shift in absorption, which may be related to the amplified distortion caused by the benzene group neighboring the 2-phenothiazine-amine, and in turn, **84** has a high molar absorptivity coefficient (1.88 × 10^4^ M^−1^ cm^−1^) compared to **83** (0.80 × 10^4^ M^−1^ cm^−1^), which could be attributed to the incorporation of the ethylene fragment reducing the steric hindrance between the donor and the auxiliary acceptor in **84**. Estimating the energy gap values (E_0-0_) of compounds **83** and **84** was found to be 1.95 eV and 2.24 eV, respectively, which is consistent with the red-shifted absorption of **83**. HOMO energy levels for **83** and **84** (Table 2, entries 1 and 2) were considerably lower than the redox potential of the electrolyte, exhibiting a viability in the regeneration of the dye, while the calculated LUMO energy levels (Table 2) suggest sufficient driving force for electron injection into the conduction band of TiO_2_. The photovoltaic efficiencies were determined in a DSSC based on iodine electrolyte, where the maximum band of the IPCE spectrum for **84** was relatively higher than that for **83**, which can be attributed to the more negative LUMO level, as well as to the presence of the ethynyl group that confers a high molar extinction coefficient. As a result, **84** exhibited a high *J_sc_*, consistent with the excellent optical capture capacity observed in the IPCE spectra. On the other hand, **83** showed a lower *V_oc_* than **84**, indicating that the presence of the benzene group suppresses the recombination of interfacial charges with the electrolyte. In summary, the addition of extra benzene and ethynyl conjugated groups in **84** serves as the isolating groups between the 2-phenothiazine-amine donor and benzothiadiazole auxiliary acceptor with the aim of suppressing the charge recombination and strengthening molecular planarity synchronously, which leads to an improvement in *J_sc_* and *V_oc_* values and contributes to a better PCE value for **84**.

**Figure 24:**
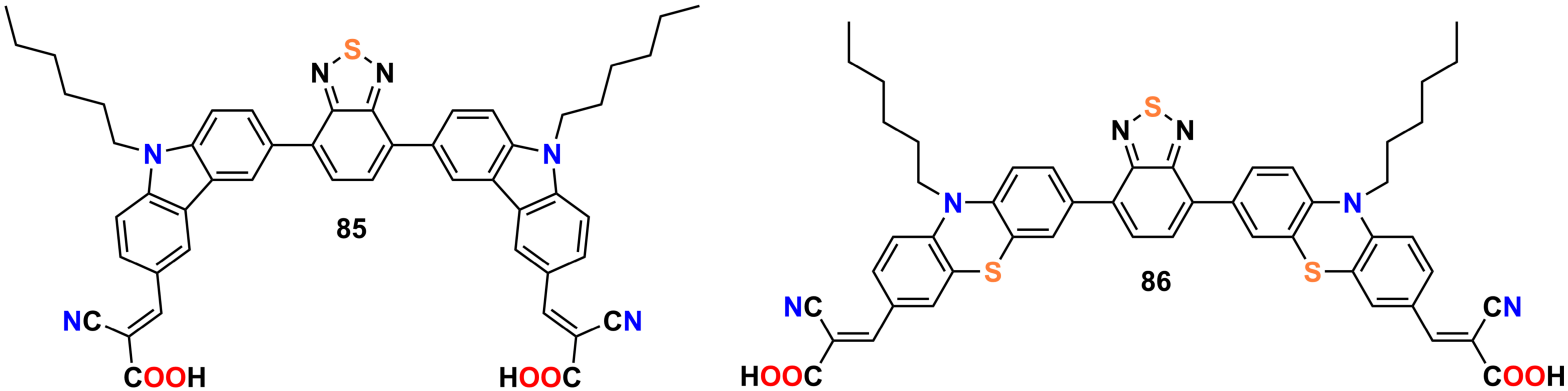
Structure of A-D-π-D-A type organic dyes for DSSCs.

Murali et al. [80] reported the synthesis of two banana-shaped dyes based on carbazole and phenothiazine linked through benzothiadiazole core **85** and **86**, respectively (Figure 24). A key feature of their design is the inclusion of two anchoring groups at both ends of the molecule to strengthen dye absorption onto the TiO_2_ semiconductor surface, enhance light-harvesting capabilities, facilitate better ICT, and ultimately improve the overall PCE of the solar cells. The dye **86** exhibited better optoelectronic properties than the carbazole dye **85** because phenothiazine is a significantly stronger electron donor, with a much broader absorption spectrum. Additionally, the butterfly geometry of the phenothiazine enhances π-conjugation. When dye **86** was incorporated into the DSSC device, a PCE of 6.87% was observed, while dye **85** showed a PCE of 4.35%. This substantial difference is driven by a massive boost in *J_sc_*, which nearly doubled due to better absorption properties. Furthermore, the non-planar geometry of the phenothiazine-based dye prevents molecular aggregation on the TiO_2_ surface, thereby enabling superior charge separation.

## 5. Conclusions and Perspectives

Solar energy technologies are proposed as an alternative to traditional non-renewable energy sources, with perovskite-based solar cells (PSCs) and dye-sensitized solar cells (DSSCs) standing out for their efficiency and low-cost materials. Phenothiazine has played a fundamental role as a starting point for the development of new structural components (e.g., hole transport layers, photosensitizers, and interfacial materials). This is thanks to the important optoelectronic properties of this core, such as good charge transport, high light capture, easily tunable absorption maxima, a butterfly-shaped molecular structure that reduces molecular aggregates, and excellent thermal and environmental stability, as well as low environmental impact. Phenothiazine is normally used as a donor unit due to the high number of electrons in its heteroaromatic fragments with sulfur and nitrogen atoms. The synthetic versatility of this molecule allows easy and efficient structural functionalization via nucleophilic substitution reactions and metal-catalyzed cross-couplings, such as Buchwald–Hartwig, Suzuki, Ullmann, Sonogashira, and others, yielding phenothiazine-based molecules with good yields (>60%). All these features enable the design of various structures to improve the photophysical and optoelectronic parameters of the compounds and devices. Phenothiazine derivatives can be used as photosensitizers in DSSCs and hole transport materials, and even as interfacial modifiers in PSCs with different types of architectures, achieving PCEs of 25.75% and 12.85% for PSCs and DSSCs, respectively, which are close to those obtained with well-established organic and inorganic reference materials (spiro-OMeTAD and N719). The advantages of phenothiazine-based devices include improved optoelectronic and photovoltaic properties, as well as exceptional solubility, hydrophobicity, and thermal stability compared to other carbazole- and phenoxazine-based materials, which could facilitate their scalability. Therefore, it is evident that phenothiazine units significantly improve the photovoltaic parameters of the cells. Despite the significant advances in phenothiazine derivatives with photovoltaic applications, challenges remain, such as (i) exploring of new, more efficient, and economical synthetic routes for the preparation of new phenothiazine-derived materials; (ii) improving of properties such as mobility and charge separation, redox potentials, physicochemical and photophysical properties, through careful molecular structure design; all with the aim of (iii) exploring new phenothiazine-based structures for use in photovoltaic devices. Based on the above, it can be concluded that phenothiazine and its derivatives are and will continue to be compounds of great interest in fields such as organic synthesis and the development of photovoltaic devices in the following years.

## Figures and Tables

**Table 2:** Compilation of the optoelectronic and photophysical properties of compounds used in DSSC ^[a]^.

Entry	Phenothiazine Derivatives	λ_max_ (nm)	E_g_ (eV)	E_ox_ (V)	*V_oc_* (V)	*J_sc_* (mA·cm^−2^)	*FF* (%)	PCE (%)	Ref.
1	52	433	1.86	0.87	0.67	16.84	70.02	8.02	[69]
2	53	429	1.87	0.82	0.65	11.45	72.76	5.41	
3	54	447	2.54	1.3	0.7	11.84	72	5.97	[70]
4	55	414	2.51	1.27	0.67	7.18	89	4.28	
5	56	259	2.27	0.78	0.78	16.66	68	8.8	[71]
6	57	260	2.07	0.76	0.75	14.7	71	7.83	
7	58	458	2.24	0.79	0.8	16.73	68	9.05	
8	59	259	2.05	0.76	0.77	14.42	71	7.9	
9	60	422	2.46	-	0.674	14.66	72.12	7.12	[72]
10	61	481	2.15	-	0.715	13.37	69.33	6.63	
11	62	488	2.09	-	0.746	15.63	71.69	8.36	
12	63	449	2.5	-	0.71	10.8	69.36	5.35	[73]
13	64	506	2.28	-	0.64	10.06	69.1	4.43	
14	65	447	2.14	-	0.81	13.33	61.96	6.69	[74]
15	66	468	2.02	-	0.82	14.04	63.67	7.33	
16	67	-	2.35	-	0.79	13.08	68	6.96	[75]
17	68	-	2.33	-	0.46	0.96	58	0.26	
18	69	-	2.42	-	-	-	-	-	
19	70	-	2.29	-	-	-	-	-	
20	71	470	2.43	-	0.69	14.25	69	6.76	[76]
21	72	496	2.29	-	0.62	11.16	71	4.84	
22	73	475	2.3	-	0.59	8.76	76	3.94	
23	74	500	2.06	-	0.825	18.96	65.27	10.21	[19]
24	75	550	1.93	-	0.866	19.98	67.61	11.7	
25	76	330	2.28	0.82	0.84	11.03	58.06	5.38	[77]
26	77	336	2.27	0.8	0.85	12.77	60.21	6.59	
27	78	334	2.23	0.77	0.82	7.99	58.53	3.83	
28	79	337	2.22	0.75	0.83	9.36	59.23	4.46	
29	80	365	2.68	-	0.72	11.46	64	5.28	[78]
30	81	417	2.65	-	0.69	12.76	66	5.75	
31	82	408	2.66	-	0.64	12.88	63	5.24	
32	83	516	1.95	0.79	0.69	8.01	61.24	3.37	[79]
33	84	478	2.24	0.76	0.71	10.28	58.35	4.24	
34	85	440	2.36	0.91	0.762	7.51	76	4.35	[80]
35	86	471	2.18	0.62	0.728	13.1	72	6.87	

^[a]^ Device parameters. *E_g_*: optical band gap is calculated according to *E_g_* = LUMO − HOMO. *V_oc_*: open-circuit voltage. *J_sc_*: short-circuit current densities. PCE: power conversion efficiency.

## Data Availability

Not applicable.
